# Comparative chloroplast genome analyses of *Amomum*: insights into evolutionary history and species identification

**DOI:** 10.1186/s12870-022-03898-x

**Published:** 2022-11-09

**Authors:** Lu Gong, Xiaoxia Ding, Wan Guan, Danchun Zhang, Jing Zhang, Junqi Bai, Wen Xu, Juan Huang, Xiaohui Qiu, Xiasheng Zheng, Danyan Zhang, Shijie Li, Zhihai Huang, He Su

**Affiliations:** 1 The Second Clinical College of Guangzhou University of Chinese Medicine, Guangzhou, Guangdong China; 2 Key Laboratory of Quality Evaluation of Chinese Medicine of the Guangdong Provincial Medical Products Administration, Guangzhou, Guangdong China; 3 Guangzhou Key Laboratory of Chirality Research on Active Components of Traditional Chinese Medicine, Guangzhou, Guangdong China; 4 Luqiao Hospital, Taizhou Enze Medical Center (Group), Taizhou, Zhejiang China; 5 Institute of Medicinal Plant Physiology and Ecology, School of Pharmaceutical Sciences, Guangzhou University of Chinese Medicine, Guangzhou, Guangdong China; 6 School of Pharmaceutical Sciences, Guangzhou University of Chinese Medicine, Guangzhou, Guangdong China

**Keywords:** *Amomum* species, Chloroplast genome, Comparative analysis, Species identification markers, Evolutionary history, Hybridization

## Abstract

**Background:**

Species in genus *Amomum* always have important medicinal and economic values. Classification of *Amomum* using morphological characters has long been a challenge because they exhibit high similarity. The main goals of this study were to mine genetic markers from cp genomes for *Amomum* species identification and discover their evolutionary history through comparative analysis.

**Results:**

Three species *Amomum villosum*, *Amomum maximum* and *Amomum longipetiolatum* were sequenced and annotated for the complete chloroplast (cp) genomes, and the cp genomes of *A. longipetiolatum* and *A. maximum* were the first reported. Three cp genomes exhibited typical quadripartite structures with 163,269-163,591 bp in length. Each genome encodes 130 functional genes including 79 protein-coding, 26 tRNAs and 3 rRNAs genes. 113-152 SSRs and 99 long repeats were identified in the three cp genomes. By designing specific primers, we amplified the highly variable loci and the mined genetic marker *ccs*A exhibited a relatively high species identification resolution in *Amomum*. The nonsynonymous and synonymous substitution ratios (Ka/Ks) in *Amomum* and *Alpinia* showed that most genes were subjected to a purifying selection. Phylogenetic analysis revealed the evolutionary relationships of *Amomum* and *Alpinia* species and proved that *Amomum* is paraphyletic. In addition, the sequenced sample of *A. villosum* was found to be a hybrid, becoming the first report of natural hybridization of this genus. Meanwhile, the high-throughput sequencing-based ITS2 analysis was proved to be an efficient tool for interspecific hybrid identification and with the help of the chloroplast genome, the hybrid parents can be also be determined.

**Conclusion:**

The comparative analysis and mined genetic markers of cp genomes were conducive to species identification and evolutionary relationships of *Amomum*.

**Supplementary Information:**

The online version contains supplementary material available at 10.1186/s12870-022-03898-x.

## Background

*Amomum* Roxb. is the second largest genus after *Alpinia* Roxb. in Zingiberaecae family, which includes approximately 150-180 species that is widely distributed in Southeast Asia [1, 2]. There are 24 species and two variants in China and most of them have medicinal and culinary values [3]. Some famous species in this genus are used as traditional Chinese medicine and included in the Chinese Pharmacopoeia: *Amomum villosum*, *Amomum villosum* var. *xanthioides*, *Amomum longiligulare*, *Amomum tsao-ko*, *Amomum kravanh* and *Amomum compactum* [4]; other 16 species of this genus are found to be important folk medicines in southeast Asian countries [5]. The plants of this genus have been usually used in the treatment of antibiotic-associated diarrhea (AAD), steatohepatitis and functional dyspepsia (FD), etc. [5]. Therefore, a great deal of studies about the phytochemistry and pharmacology of the plants from *Amomum* genus have been carried out [5]. Among these species, *A. villosum* is one of the authentic species of a famous traditional Chinese medicine Amomi Fructus. Higher active ingredient content of borenol acetate in the fruit of *A. villosum* than in *A. villosum* var. *xanthioides* and *A. longiligulare* was confirmed by studies, which is the presumable cause of better therapeutic effect of *A. villosum* [6]. *Amomum maximum* Roxb. rhizome is a folk medicine mainly used in South and Southeast Asia [7]. *Amomum longipetiolatum* is distributed in Guangxi and Hainan provinces of China and the neighboring country Vietnam, which is not studied that much.

Classification of *Amomum* using morphological characters has long been a challenge as they exhibit high similarity among different species in this genus [1]. This problem also leads to the confusion of *Amomum* species in commercial herbal market, and authentic species are often misidentified; for example, the ripe fruits of *A. villosum* var. *xanthioides*, *A. longiligulare* and other species from *Amomum* or even *Alpinia* have been adulterated or substituted for *A. villosum* during the sales process [8, 9]. And this phenomenon severely hinders the clinical use and scientific research of *Amomum* species. Previous studies have conducted the molecular classification and identification of *Amomum* species. Shi et al. [10] evaluated eight candidate DNA barcodes, finding ITS/ITS2 were more suitable for *Amomum* species identification. And ITS proved to be able to differentiate Amomi Fructus from its adulterants, however, it couldn’t distinguish the three authentic plant species [9]. Based on the analysis of ITS and *mat*K, Xia [11] found that *Amomum* was polyphyletic with three major groups of species. Further, de Boer [1] recircumscribed the 9 clades of the paraphyletic *Amomum* as a monophyletic genus by combining molecular data and morphological characters. These results indicated that more informative sites were needed to determine the relationships among species and accurately identifying species within the genus.

The chloroplast (cp) is an important organelle providing essential energy for plants, which mainly conduct photosynthesis that converts solar energy into chemical energy and releases oxygen. The cp has its own genome comprising a circular DNA that is independent from nuclear one. In angiosperms, cp genomes are highly conserved in gene content and genome structure and usually uniparental inheritance, and have low nucleotide substitution mutation rates [12]. With the improvement of high throughput sequencing, they are more conveniently obtained and widely used for species identification [13] and investigation of phylogenetic evolution [14, 15] in recent years. Although there has been over 2200 complete plant cp-genomes deposited in the public database by 2021 [16], it’s still insufficient and only cp genomes of six species included in the Chinese Pharmacopoeia for *Amomum* are publicly available at present. And these *Amomum* species with cp genomes published were all in the villosum and tsao-ko groups according to Xia’s study [11]. Here, we sequnced and anotated cp genomes of *A. maximum* and *A. longipetiolatum* in the other maximum group besides of *A. villosum* in the villosum group, then conducted phylogenetic analysis to evaluate plant relationships among *Amomum* and even Zingiberaceae.

Most studies of cp genomes explored the variable regions for species identification by bioinformatics analysis [17], whereas, more and more studies selected and tested the specific barcodes through experiments verification in recent years. Scanning cp genomes for more variable markers has improved identification success in several plant groups [18–20]. For species in Zingiberaecae, Cui et al. [8] sequenced and annotated cp genomes of *A. villosum*, *A. villosum* var. *xanthioides* and *A. longiligulare*. However, they found that highly divergent regions screened from cp genomes could not be used to distinguish *Amomum* species. Zhang et al. [21] reported the cp genomes of *Alpinia galanga* and *Alpinia kwangsiensis*. Finally, five candidate markers were found for species-level identification of *Alpinia* through interspecific comparisons. In this study, we explored the highly divergent regions of cp genomes for *Amomum* species identification using different species from those in Cui’s study [8].

Overall, we report the complete cp genomes of *A. villosum*, *A. longipetiolatum* and *A. maximum* in this study. Then, we conducted comparative analysis among these cp genomes with other published cp genomes in *Amomum*. Our main objectives were: (1) to explore the molecular structures of three chloroplast genomes; (2) to examine the repeat sequences among *Amomum* chloroplast genomes; (3) to discover sequence variations and highly divergent regions, and select specific DNA markers for *Amomum* species identification; (4) to assess the evolutionary history and explore the phylogenetic relationships of *Amomum*.

## Results

### Chloroplast genome features and organizations

**Fig. 1:**
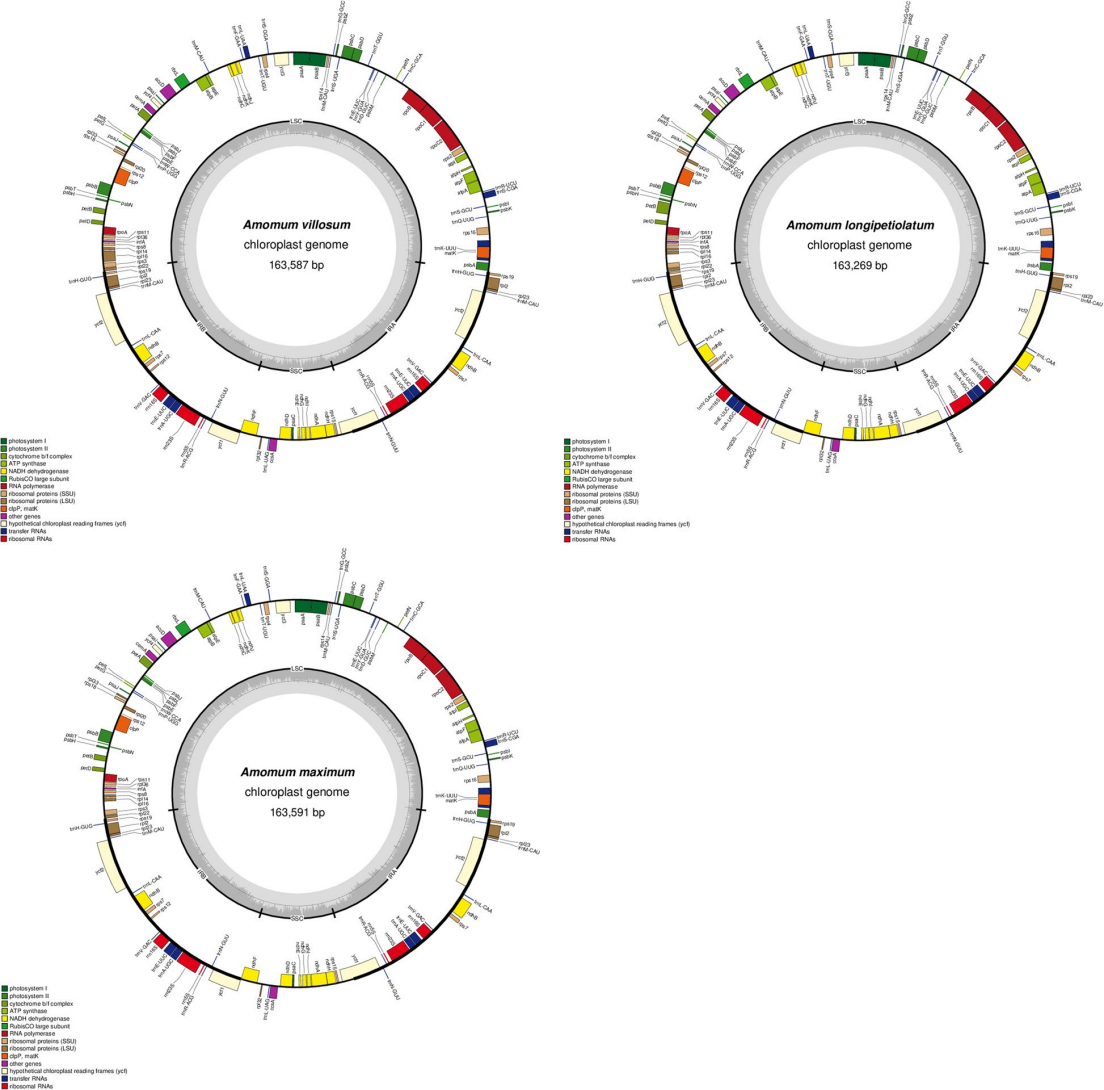
Chloroplast (cp) genome maps of *A. villosum*, *A. longipetiolatum* and *A. maximum.* Genes inside the circle are transcribed clockwise, and those outside are transcribed counter-clockwise. Genes in different functional groups are color-coded. The small (SSC) and large (LSC) single copy regions and inverted repeat (IRa and IRb) regions are noted in the inner circle

Fresh samples of *A. villosum*, *A. longipetiolatum* and *A. maximum* were used as source for gDNA, and subjected to NGS with Illumina NovaSeq paired-end sequencing. Chloroplast-like sequences were extracted from clean Ilumina reads, by BLAST searches against an in-house constructed chloroplast database, assembled with SOAPdenovo [22] and annotated with CPGAVAS2 [23]. De novo assembled chloroplast genomes were deposited in GenBank with accession numbers (*A. villosum* MW995976, *A. longipetiolatum* MW970344, and *A. maximum* MW995975). The complete cp genomes of three species ranged from 163,269 bp to 163,591 bp in size and exhibited typical quadripartite structures (Fig. 1). The overall GC content for these three species was nearly identical (36.08-36.13%) but was unevenly distributed in the cp genomes (Table 1). In details, the GC content was the highest in IR regions (41.15-41.17%) while the lowest in SSC regions (29.82-30.25%). Furthermore, AT appearance at the third codon position (71.14-72.83%) was higher than that at the first (54.86-55.41%) and second (61.98-62.62%) positions in the protein-coding regions (CDS) of three species. The complete cp genomes of three species encode 130 functional genes. After removing duplicates, 108 unique genes including 79 protein-coding, 26 tRNAs and 3 rRNAs genes were remained for each genome (Table 2). Among these genes, 19 genes were duplicated in IR regions. There were 16 genes containing introns for each cp genomes, and 15 genes were shared among all the species. *Pet*B, *rpl*16 and *ycf*1 were distinct intron-containing genes for *A. villosum*, *A. longipetiolatum* and *A. maximum* respectively. For the 16 genes, 14 genes contained one intron, and the other two genes *ycf*3 and *clp*P contained two introns. It’s worthy to note that *trn*K*-UUU* gene had the longest intron more than 2500 bp.

**Table 1:** Chloroplast genome features of *A. villosum, A. longipetiolatum* and *A. maximum*

Type	*A.villosum*	*A.longipetiolatum*	*A.maximum*
Total Length (bp)	163,587	163,269	163,591
LSC length (bp)	88,659	88,461	88,615
IR length (bp)	29,822	29,665	29,775
SSC length (bp)	15,284	15,478	15,426
CDS length (bp)	81,372	82,740	69,111
Number of genes	130	130	130
Protein-coding genes	87	87	87
rRNA genes	37	37	37
tRNA genes	6	6	6
Total GC content	36.08%	36.13%	36.13%
GC content of LSC	33.71%	33.77%	33.84%
GC content of IRa	41.15%	41.17%	41.17%
GC content of IRb	41.15%	41.17%	41.17%
GC content of SSC	30.06%	30.25%	29.82%
CDS.AT1	55.16%	55.41%	54.86%
CDS.AT2	62.36%	62.62%	61.98%
CDS.AT3	71.14%	71.17%	72.83%

**Table 2:** Genes encoded in the cp genomes of *A. villosum, A. longipetiolatum* and *A. maximum*

Category of genes	Group of genes
photosystem I	*psa*A, *psa*B, *psa*C, *psa*I, *psa*J
photosystem II	*psb*A, *psb*B, *psb*C, *psb*D, *psb*E, *psb*F, *psb*H, *psb*I, *psb*J,
	*psb*K, *psb*L, *psb*M, *psb*N, *psb*T, *psb*Z, *ycf*3**
cytochrome b/f complex	*pet*A, *pet*B^a^, *pet*D, *pet*G, *pet*L, *pet*N
ATP synthase	*atp*A, *atp*B, *atp*E, *atp*F*, *atp*H, *atp*I
NADH-dehydrogenase	*ndh*A*, *ndh*B (×2)*, *ndh*C, *ndh*D, *ndh*E, *ndh*F, *ndh*G, *ndh*H, *ndh*I, *ndh*J, *ndh*K
rubisco	*rbc*L
DNA dependent RNA polymerase	*rpo*A, *rpo*B, *rpo*C1*, *rpo*C2
Small subunit of ribosome	*rps*2, *rps*3, *rps*4, *rps*7 (×2), *rps*8, *rps*11, *rps*12 (× 2),
	*rps*14, *rps*15, *rps*16*, *rps*18, *rps*19 (×2),
	*rpl*2 (×2)*, *rpl*14, *rpl*16^b^, *rpl*20, *rpl*22, *rpl*23 (× 2), *rpl*32, *rpl*33, *rpl*36
Conserved open reading frames	*ycf*1^c^ (×2), *ycf*2 (× 2), *ycf*4
Ribosomal RNAs	*rrn*5 (×2), *rrn*16 (× 2), *rrn*23 (× 2)
Transfer RNAs	*trn*A*-UGC* (×2)*, *trn*C-GCA*, *trn*D*-GUC*, *trn*E*-UUC* (×3)*, *trn*F*-GAA*, *trn*G*-GCC*, *trn*H*-GUG* (× 2), *trn*K-UUU*, *trn*L*-CAA* (× 2), *trn*L-UAA*,
	t*rn*L*-UAG*, t*rn*M*-CAU* (×4), *trn*N*-GUU* (×2), *trn*P*-UGG*, *trn*Q*-UUG*,
	*trn*R*-ACG*(×2), *trn*R*-UCU*, *trn*S*-GCU*, *trn*S*-GGA*, *trn*S-CGA*, *trn*S*-UGA*, *trn*T*-UGU*, *trn*T*-GGU*, *trn*V*-GAC* (× 2), *trn*W*-CCA*, *trn*Y*-GUA*
Other genes	*acc*D*, *ccs*A, *cem*A, *clp*P **, *inf*A, *mat*K

(×2) indicates the gene sequence is repeated twice. * indicates genes containing one intron; while ** indicates gene containing two introns. a, b and c was contained an intron in *A. villosum*, *A. longipetiolatum* and *A. maximum* respectively

### Codon usage and repeat content

Amino acids frequency and codon usage were determined for *A. villosum*, *A. longipetiolatum* and *A. maximum* in CodonW. All the protein-coding genes were composed of 23,037–27,580 codons in the three cp genomes. Just as in most angiosperms [24], leucine was the most abundant amino acid in three *Amomum* species with a frequency of 10.22–10.26%, followed by isoleucine (8.55–8.61%).

The type of SSRs and its distribution in cp genomes were analyzed by MISA. 152, 113 and 130 SSRs were identified for *A. villosum*, *A. longipetiolatum* and *A. maximum*, respectively (Fig. S1 and Table S1). The majority of SSRs were mono-nucleotide followed by di- and tetra-. Interestingly, mono-, di-, tri-, tetra- and penta-nucleotide repeats were all detected in the three cp genomes, while hexa- nucleotides only occured in *A. villosum*. Most of the mononucleotide SSRs consisted of A/T motifs that enriching A and T in the cp genomes, and they were the most frequently used base among all SSR types as previous studies confirmed [25, 26]. However, number of A/T repeats differed among three species, ranging from 50 in *A. longipetiolatum* to 90 in *A. villosum* (Fig. S2). Other two cp genomes of *A. kravanh* (MF991963) and *A. compactum* (MG000589) were downloaded from public database to be analyzed together for the four types of long repeats. The results showed that 99 repeats exist in each of the five cp genomes. Though the number of four types’ long repeats differed among species, the palindrome repeats were found to be the most abundant while complement repeats were the least. The majority of these repeats were between 21 and 30 bp. And it’s found that only repeats in *A. maximum* were no longer than 50 bp. Moreover, the length of all the complement repeats was less than 30 bp in these cp genomes (Fig. S3).

### Boundary regions and interspecific comparisons

**Fig. 2:**
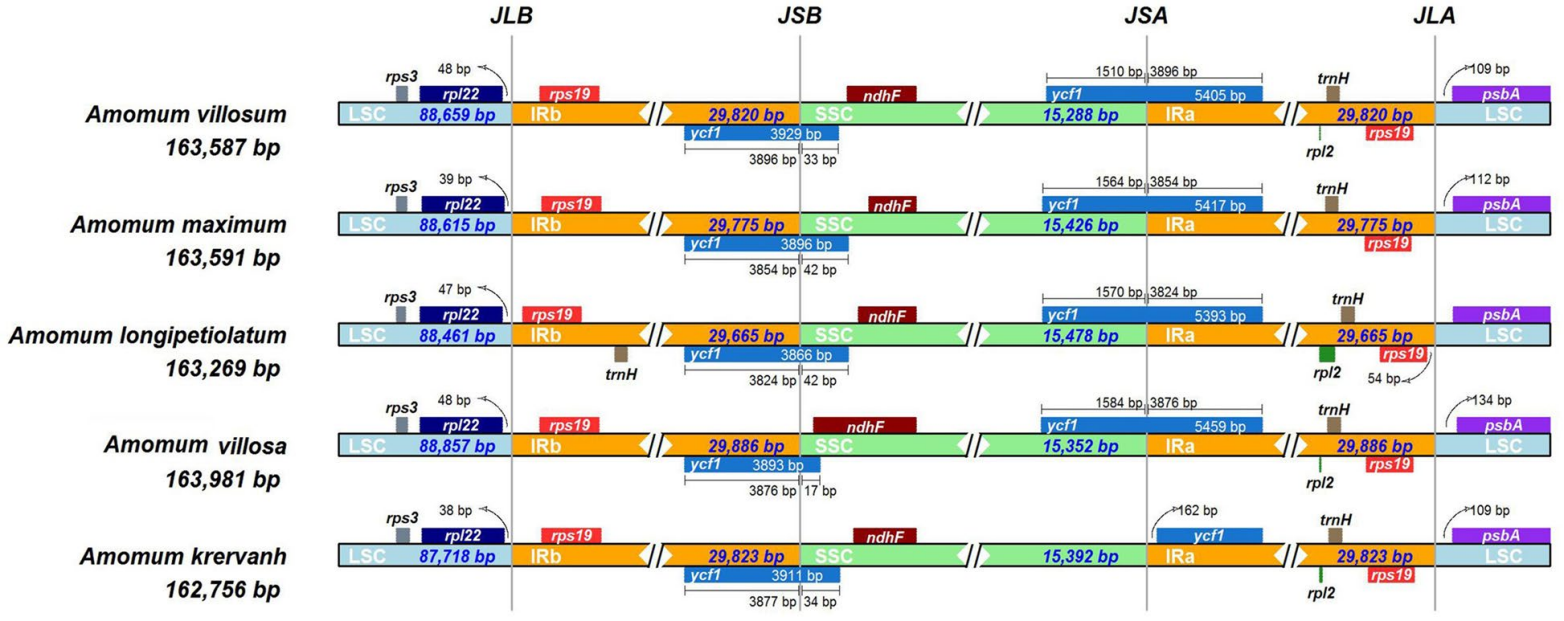
Comparison of the borders of the LSC, SSC, and IR regions among five chloroplast genomes of *Amomum*

We compared the contraction and expansion of IRs regions at four junctions between the two IRs (IRa and IRb) and the two single–copy regions (LSC and SSC) among five species of *Amomum* genus (Fig. 2). After IRscope analysis [27], IR/LSC junction of IRb was found to be located between *rpl*2 and *rps*19 with 38-48 bp away from *rpl*22. Junction of SSC/IRb (JSB) was located in partial *ycf*1 gene which ranged from 3866 to 3929 bp in IR and the remaining part of *ycf*1 gene expanded into the SSC regions from 17 to 42 bp. Meanwhile, the *rps*19 and *psb*A genes were located on side of the junctions of the IRa/LSC regions in five chloroplast genomes. These data indicated that the contractions and expansions of the IR regions exhibited relatively stable patterns with slight variations in these *Amomum* cp genomes.

**Fig. 3:**
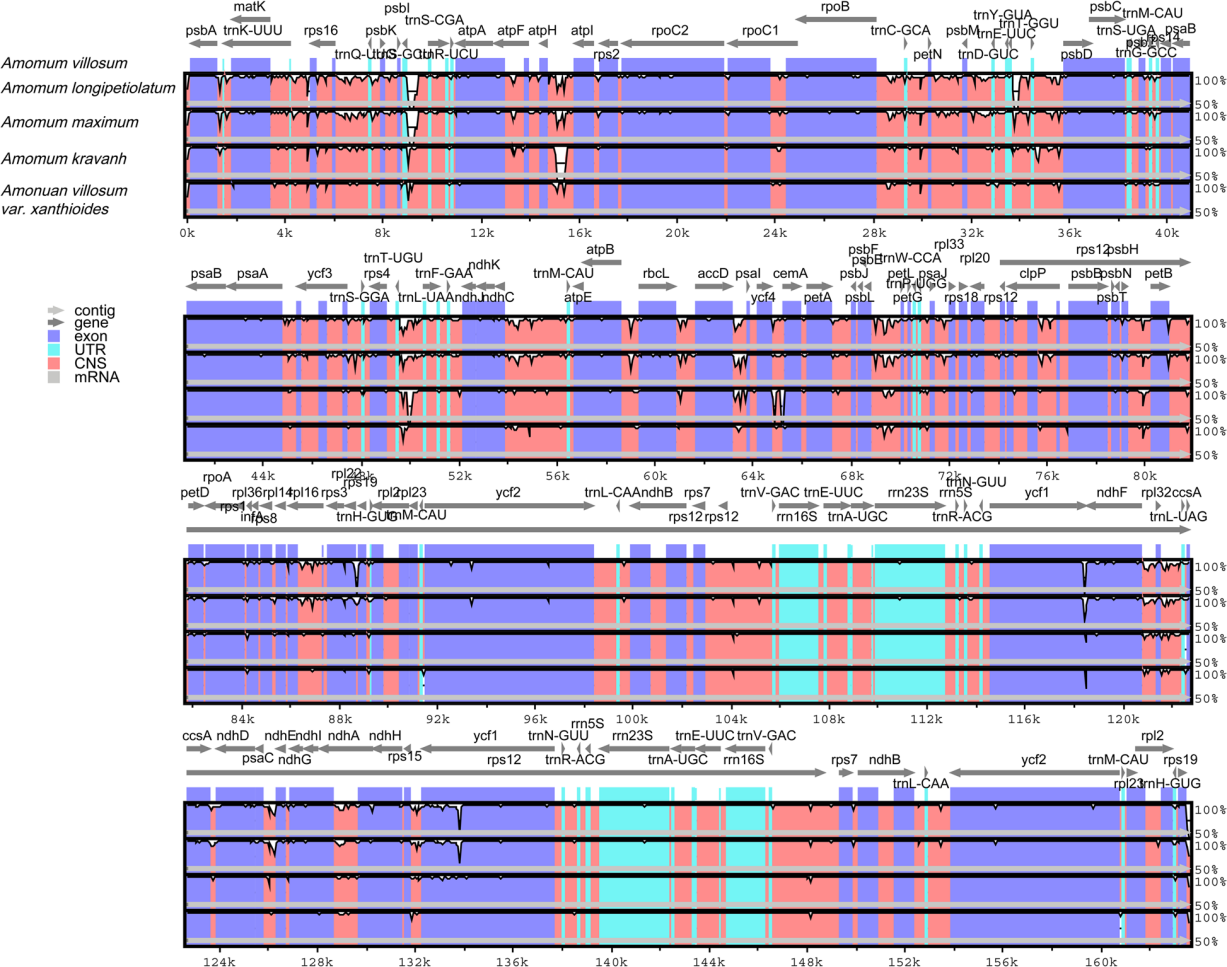
The interspecific comparisons of the five *Amomum* chloroplast genomes in mVISTA. The vertical axis indicates the percentage of identity, ranging from 50 to 100%, while the horizontal axis shows the position within the cp genome. Gray arrows display the gene orientation. Genome regions are color-coded as exon, tRNA/rRNA, CNS and mRNA. A cut-off of 70% identity was used for the plots

**Fig. 4:**
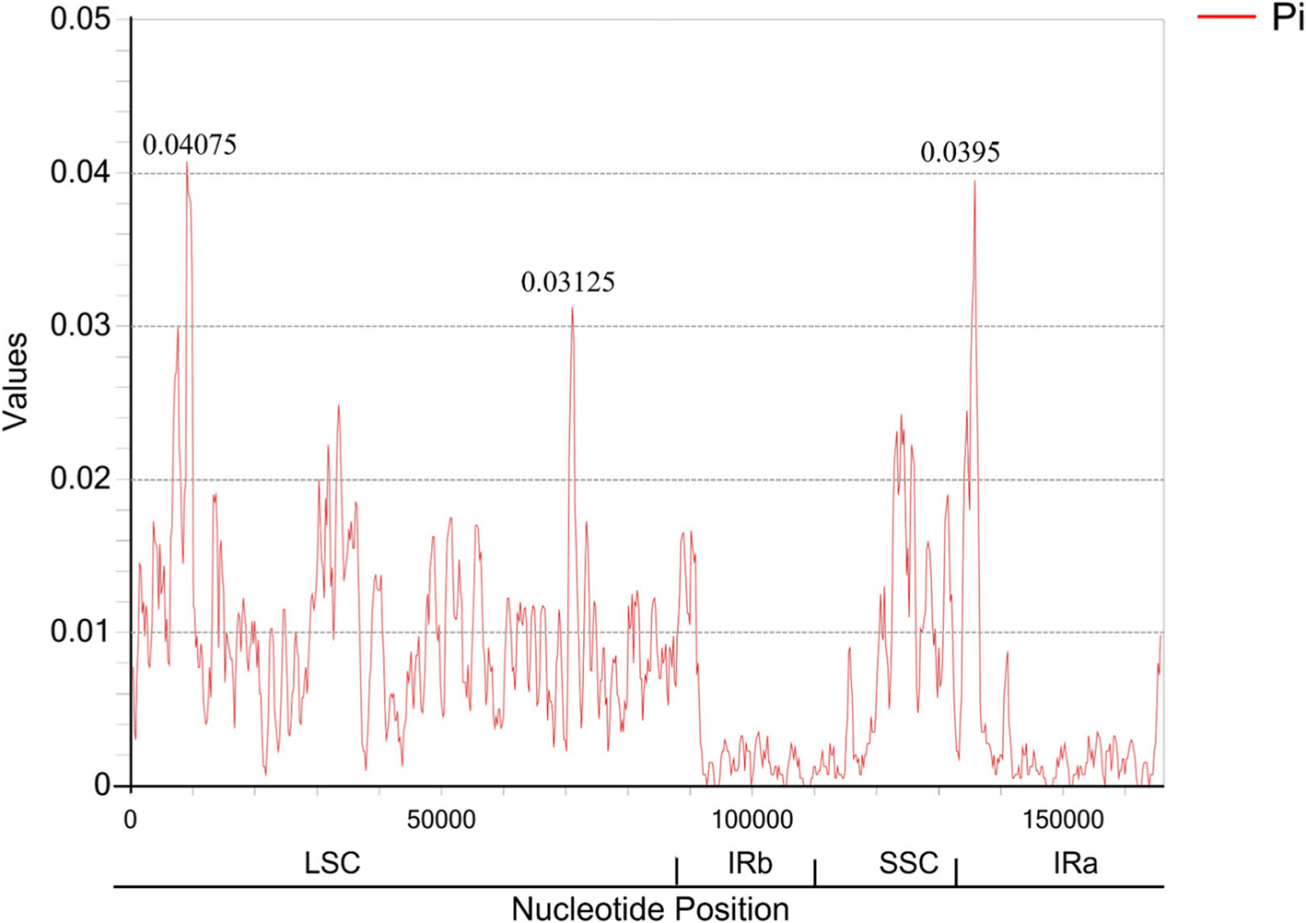
Sliding window analysis of the five *Amomum* cp genomes. Window length: 800 bp; step size: 200 bp. X-axis: position of the midpoint of a window. Y-axis: nucleotide diversity of each window

In order to compare the sequence variations within the *Amomum* genus, complete cp genomes of five *Amomum* species were aligned using the program mVISTA with *A. villosum* as a reference (Fig. 3). Among the 5 taxa, *A. villosum* and *A. villosum* var. *xanthioides* showed great similarity with each other in the genetic divergence. More variations were discovered in the LSC and SSC regions than IR regions. The most highly divergent regions among the 5 cp genomes were in intergenic spacers regions. Furthermore, sliding window analysis revealed highly variable regions in the 5 *Amomum* cp genomes. As shown in Fig. 4, mutational hotspots within these *Amomum* species were commonly located in the LSC and the SSC regions, which was consistent with the result of mVISTA. The nucleotide diversity (Pi) values were calculated with DnaSP [28] to test divergence level within different regions among the 5 *Amomum* cp genomes. The average value of nucleotide diversity (Pi) was 0.00702.

### Species identification markers mining

To develop new identification markers of *Amomum*, 70 shared protein-coding genes and 44 shared intergenic regions of the five *Amomum* species were extracted for nucleotide diversity (Pi values). The results showed that nucleotide diversity of the *Amomum* species ranged from 0.00000 to 0.04075, with a mean of 0.00752. 16 CDS and 22 intergenic regions with high Pi values were then aligned and designed with specific primer pairs as candidate markers. 37 leaf samples of 13 *Amomum* species were collected and used to test the selected markers by PCR and Sanger sequencing. PCR efficiency and species identification resolution through ML tree were used as evaluation indicators. Finally, 11 markers were obtained with high amplification efficiency (89.19-100%) and sequence quality using 37 samples of 13 *Amomum* species (Table S2). As a result, *ccs*A showed the strongest discrimination power for *Amomum* species among these mined markers based on phylogenic analysis, followed by *trn*C*-GCA_pet*N, *rpl*20, *rps*3 and *rpo*A (Fig. S4-S14).

For comparison, we also amplified and sequenced the plant universal barcodes (ITS/ITS2, *mat*K, *rbc*L and *psb*A-*trn*H) [29] from the 37 samples. Among all the DNA barcodes, *mat*K had the lowest PCR success rates of 27.03% that was not suitable for *Amomum* species identification (Table 3). From the ML tree, the other two universal markers from cp genomes *rbc*L and *psb*A*-trn*H with higher PCR success rates than *mat*K showed lower species discrimination efficiency (Fig. S15-S19) than most of the mined markers other than *rpl*33 and *ndh*B*_rps*7. Thus, the mined specific markers always performed a better species identification resolution and a higher PCR efficiency than the universal markers *mat*K, *rbc*L and *psb*A*-trn*H from cp genomes. Therefore, these mined markers, especially *ccs*A, could be considered as the candidate DNA barcodes for *Amomum* species identification. More importantly, we found that the mined marker *trn*C*-GCA_pet*N have the potential to distinguish *A. villosum* from *A. villosum* var. *xanthioides*, as sequences of *A. villosum* var. *xanthioides* clustered into one clade that apart from those of *A. villosum*, though the bootstrap value was not that high (Fig. S5). However, we also noted that the nuclear markers ITS2 was more suitable for *Amomum* species identification than cp markers (Fig. S16 and Table 3). The characteristics of DNA markers were summarized in Table 3.

**Table 3:** Efficiency of PCR and sequence characterization of DNA barcodes

DNA barcodes	No. of sequences	PCR success rates (%)	Aligned length (bp)	Number	Variable sites	K2P	Species identification success rates ^a^ (%)
					Number (%)		
ITS2	36	97.30	236	87	36.86	0.000-0.411	76.92%
ITS	36	97.30	682	174	25.51	0.000-0.046	38.46%
*mat*K	10	27.03	725	429	59.17	0.000-1.168	15.38%
*rbc*L	29	78.38	657	15	2.28	0.000-0.018	23.08%
*psb*A*-trn*H	33	89.19	797	469	58.85	0.000-1.124	23.08%
*ndh*B*_rps*7	37	100	297	2	6.73	0.000-0.004	0
*psa*I*_ycf*4	37	100	342	12	3.51	0.000-0.018	23.08%
*trn*C*-GCA_pet*N	37	100	507	251	49.51	0.000-0.768	30.77%
*rpl*20	36	97.30	297	21	7.07	0.000-0.026	30.77%
*rpl*33	36	97.30	148	5	3.38	0.000-0.035	0
*ccs*A	34	91.89	939	33	3.51	0.000-0.023	38.46%
*rps*3	34	91.89	589	83	14.09	0.000-0.090	30.77%
*rpo*A	33	89.19	907	62	6.84	0.000-0.021	30.77%
*rps*4	36	97.30	599	40	6.68	0.000-0.025	23.08%
*ndh*D_1	35	94.59	681	18	2.64	0.000-0.020	15.38%
*ndh*D_*2*	34	91.89	645	44	6.82	0.000-0.025	23.08%

^a^ When the accessions of one species were clustered in one clade in the ML tree, it was considered successfully identified. Species identification success rates were the number of successfully identified species divided by the total number of detected species (13)

### Selective pressure analyses

**Fig. 5:**
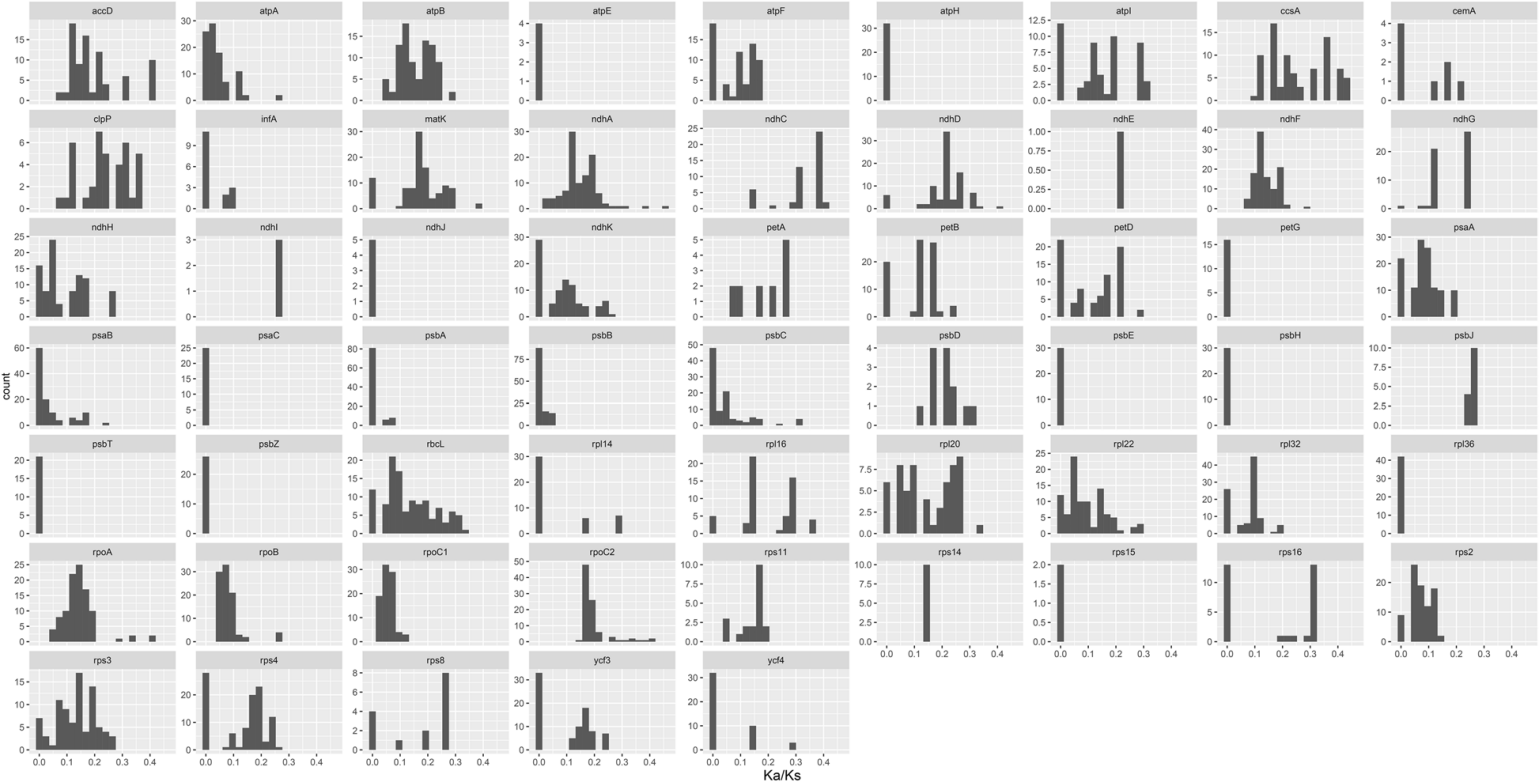
Pairwise Ka/Ks ratios for sharednon-redundant genes from *Amomum* and *Alpinia*, genes with ≥1 substitutions and significant different Ka and Ks values, examined by Fisher Exact Test by KaKs calculator were plotted. Genes like *ndhJ* only have 3 Ka/Ks values indicating most of them among different species are too conserved to calculate out the Ka/Ks values

We calculated the nonsynonymous (Ka) and synonymous (Ks) substitution ratios (Ka/Ks) for all the 70 protein coding genes of cp genomes from 12 *Amomum* as well as 5 *Alpinia* species respectively with KaKs_calculator by ‘MA’ model and statistically tested by Fisher Exact Test [30] (Table S3). We found all genes with Ka/Ks > 1 that were supposed to be positively-selected genes only have 1 substitution in the multiple sequence alignment (MSA) files for each gene-pair, because those positively-selected results all passed the Fisher Exact Test (Table S4), we further tested whether these mutations could introduce changes in 3D-structure for those genes. In detail, a total of 59 chloroplast genes with 1 substitution were found, wherein, 32 genes with both positive selection and negative selection result, 22 genes with only negative selection and 5 genes with only positive selection result (Table S4). As there are 136 combinations (choose 2 from 17) for each genes, we selected genes ≥13 occurrences with Ka/Ks > 1 (*psa*I, *psb*I, *psb*J, *inf*A, *atp*I, *cem*A, *nhd*C, *psb*D, *ndh*K) and the 5 genes (*acc*D, *mat*K, *ndh*A, *psa*I, *rps*8) with Ka/Ks values all > 1 to inspect their 3D-structures with SWISS-MODEL [31, 32] in order to test whether the mutation is functionally conserved. As a result, the majority of these genes are still conserved in 3D-structure except for *psb*I*, mat*K*, rps*8 *and psb*J (Fig. S20), indicating the majority of genes evidenced as positively selected may be false positive results. As a result, genes with ≤1 substitution were filtered out in display of Ka/Ks distribution in order to minimize the false positive results. Overall, Ka/Ks values were less than 0.5 for the majority genes, suggesting that CP genes of the *Amomum* and *Alpinia* species are conserved and mainly under a purifying selection during the evolution process (Fig. 5), which is reasonable for necessary functions played by the chloroplast genes and is in accordance with previous studies [33].

### Phylogenetic analysis

**Fig. 6:**
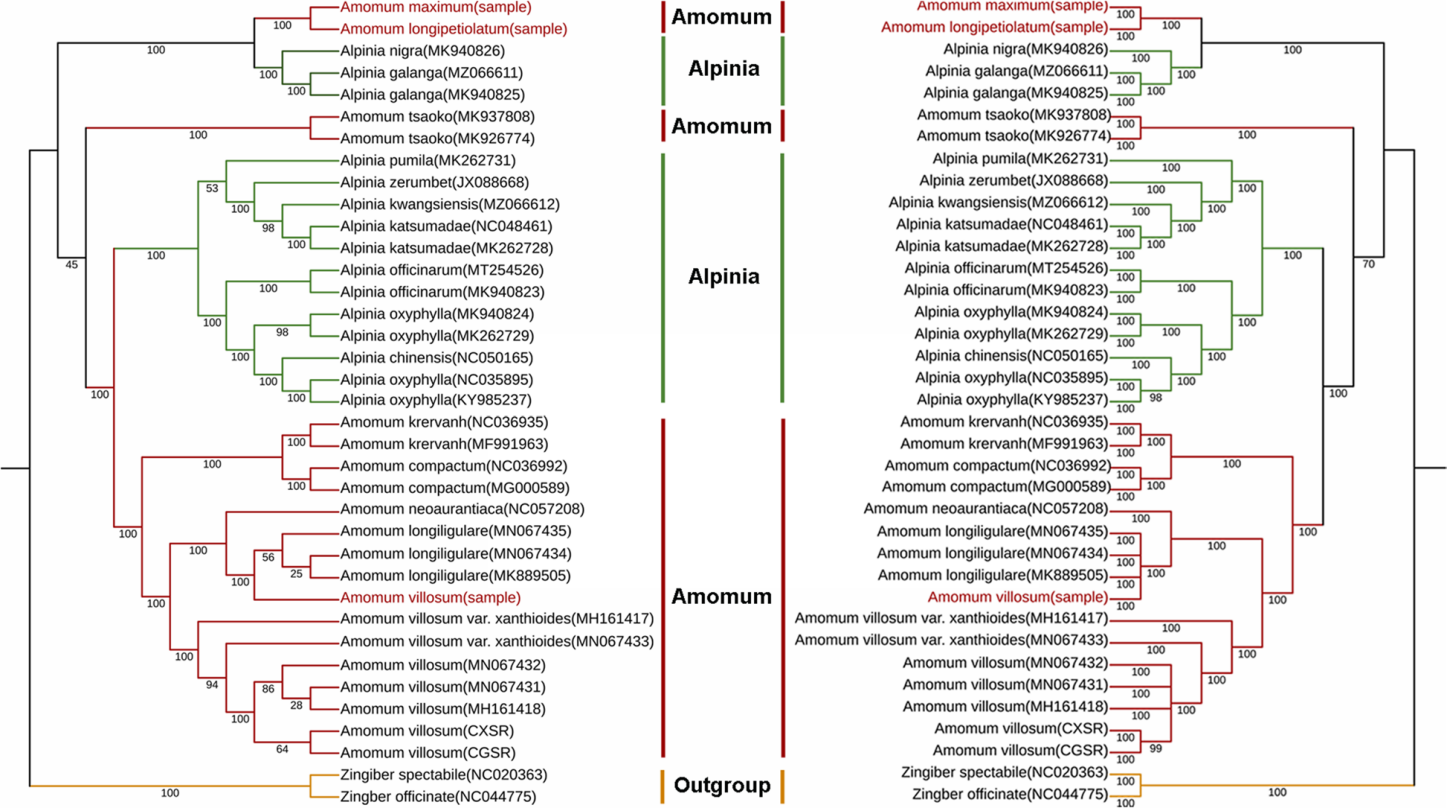
Phylogenetic trees constructed by ML (**a**) and BI (**b**) based on complete chloroplast genome. Numbers under the nodes indicate bootstrap probabilities (%) in the ML tree and post probabilities (%) in the BI tree. Our sequenced samples are marked in red. Species from different genuses are marked in different color on the nodes

To discuss the phylogenetic and evolutionary relationships of *Amomum* species, phylogenetic trees of 37 complete cp genome sequences from Alpinieae, the largest tribes of Zingiberaecae [21], were aligned by MAFFT and then constructed the phylogenetic trees by different methods including Bayesian inference (BI), maximum likelihood (ML) and neighbor joining (NJ) with the software RAxML-ng [34]. Two species from genus *Zingiber* were set up as the out-group. The results showed that most nodes in the phylogenetic trees were strongly supported (post probability or bootstrap values > 90%) except for a few ones (Fig. 6 and S21). All the BI, ML and NJ trees constructed by different methods were showing consistent topologic structures: species from *Amomum* and *Alpinia* were clustered into a paraphyletic branch. The cp genomes of *A. longipetiolatum* and *A. maximum* were used in phylogenetic analysis for the first time and they clustered together, showing a very close kinship with each other. These two species were then clustered with *Alpinia nigra* and *Alpinia galanga* as a sister group to the rest species forming another group. Next, the two sequences of *Lanxangia tsaoko* split off into another branch, and the remaining *Alpinia* and *Amomum* species split off into a large branch. In the large branch, *Alpinia* and *Amomum* species clustered into two branches, respectively. For the *Amomum* branch, *A. kravanh* and *A. compactum* first formed one clade and all the accessions of *A. villosum*, *A. villosum* var. *xanthioides* and *A. longiligulare* clustered into another clade, indicating that these three authentic plant sources of Amomi Fructus were indeed closely related. In general, our result proved that *Amomum* is paraphyletic.

### ITS2 analysis

**Fig. 7:**
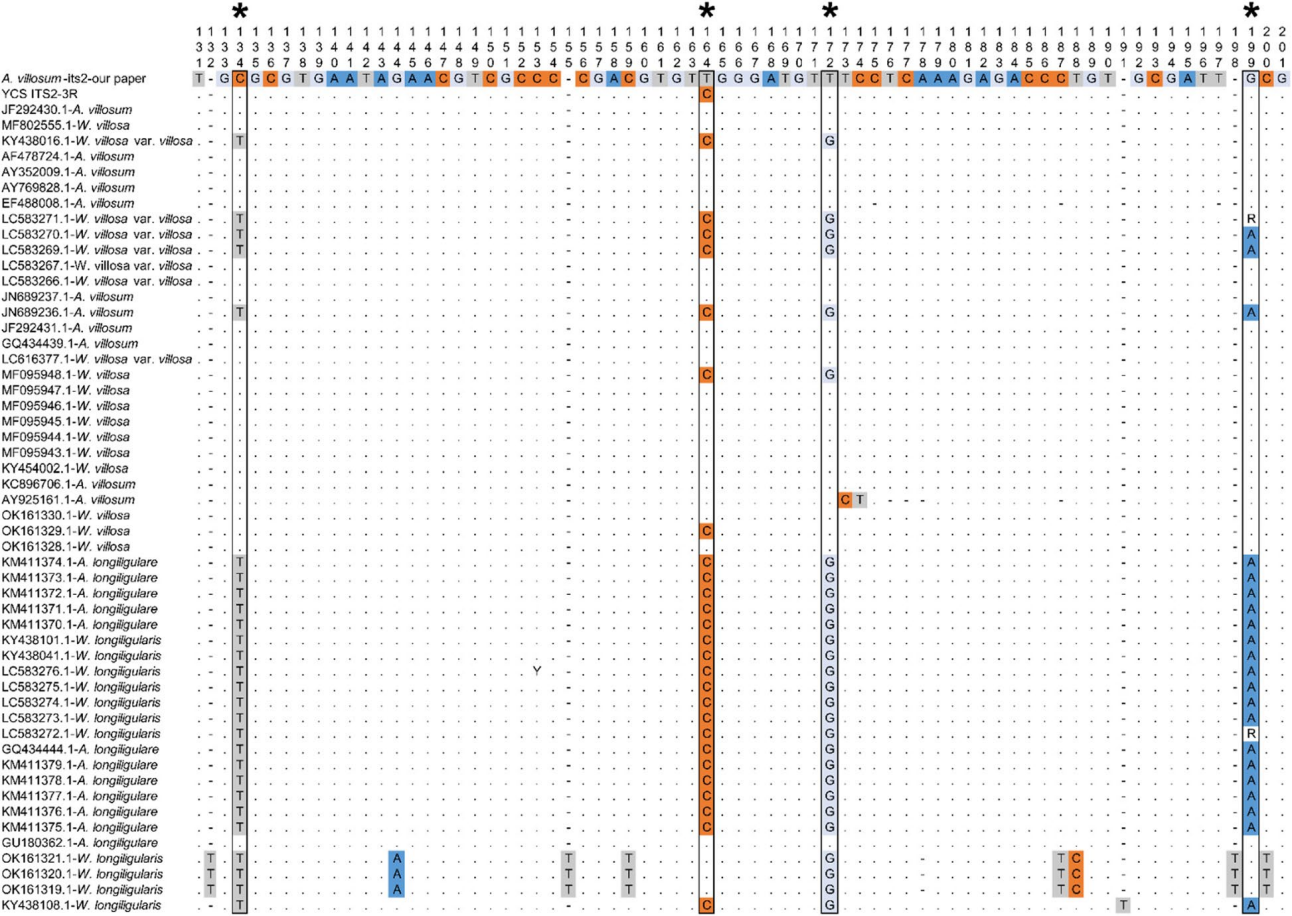
Part of ITS2 alignment of *A. villosum* and *A. longiligulare* from Sanger sequencing. Nucleotides the same as the reference are marked in dots (.) and gaps are marked in dashes (−)

**Fig. 8:**
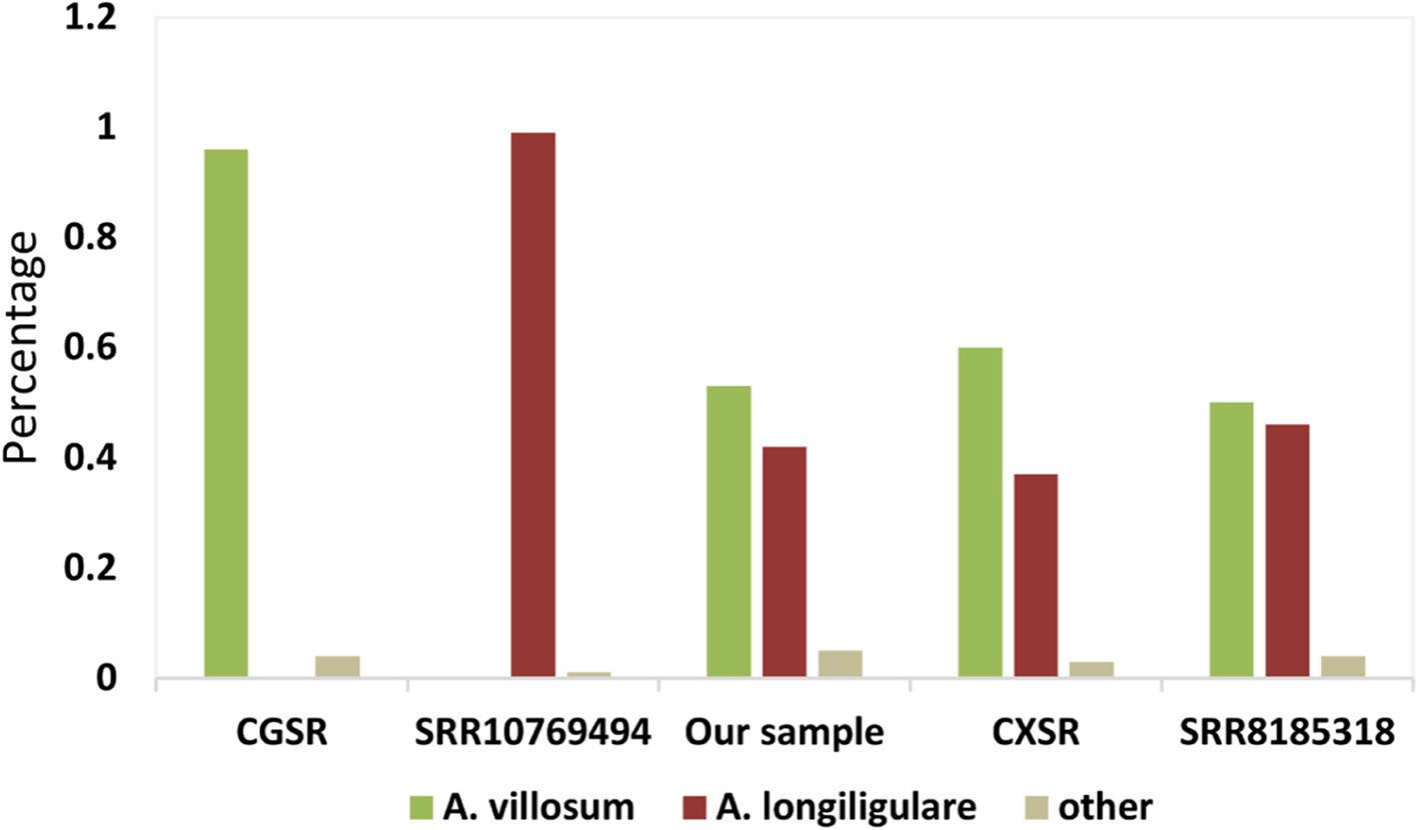
ITS2 haplotype compositions of next-generation sequencing data of *A. villosum* and *A. longiligulare*. This histogram shows the haplotype composition of ITS2 reads. The former two samples are non-hybrids and the latter three samples are hybrids. When the ITS2 reads cannot match the haplotypes of *A. villosum* or *A. longiligulare* in the Sanger sequencing analysis, they are classified as other types

Unusually, we noticed that our sample of *A. villosum* clustered with samples of *A. longiligulare* into a clade, which was separated with other samples of *A. villosum* in the phylogenetic analysis*.* We inferred that the collected sample of *A. villosum* in our study is possibly a hybrid with the female parent of *A. longiligulare* and the male parent of *A. villosum*, as the cp genome is always maternal inherited. We investigated the gene fragment of nuclear genome for more evidence. We aligned the sequencing reads of NGS data to the ITS2 sequence of *A. villosum* with Bowtie2 [35]. Consequently, four nucleotide sites were found SNP sites though IGV [36, 37]. Further, we downloaded all the Sanger sequencing data of ITS2 sequences of *A. villosum* and *A. longiligulare* from NCBI and aligned the sequences with MEGA. Then the ITS2 haplotypes of *A. villosum* and *A. longiligulare* were summarized based on the four sites (Fig. 7 and Table S5). As a result, some species-specific loci of these two species both appeared among ITS2 reads in the NGS data of our *A. villosum* (Fig. 8 and Table S6), providing another hybrid evidence of this sample.

For validation, we also analyzed other NGS data of *A. villosum* and *A. longiligulare*. Finally, we found species-specific loci in each of the *A. villosum* (CGSR) and *A. longiligulare* (SRR10769494) sample, whereas, similar to our sample, there were species-specific loci of both two species in the artificially hybridized sample of *A. villosum* (CXSR, Fig. 8 and Table S6) [38]. Interestingly, we found another sample of *A. villosum* (SRR8185318) hybridized through our ITS2 analysis (Fig. 8 and Table S6) and the published phylogenetic analysis [39], which further proved the reliability of the method. To our knowledge, this is a novel method for hybridized species identification and the first report of natural hybridizations of *Amomum*.

## Discussion

### Chloroplast genome evolution of *Amomum*

In this study, we sequenced and annotated complete cp genomes of *A. villosum, A. longipetiolatum* and *A. maximum*. The three cp genomes exhibited typical quadripartite structures. The features of these three cp genomes were very conserved among *Amomum* species [8, 40, 41] and even species from family Zingiberaceae [42, 43]. The higher GC content of the IR region than the LSC and SSC regions could be due to a reduced number of duplicated AT nucleotides in the ribosomal RNA (rRNA) genes (Fig. 1) [44] and this might be one of the causes for the IR region to be more conserved than LSC and SSC regions [45]. This phenomenon is also evident in many other angiosperms [46, 47]. There are 79 protein-coding genes in the three *Amomum* plastomes, meanwhile, 70 up to 88 such genes are reported present in angiosperm plastomes [48]. Similar to other cp genomes, the A/T appearance at the third codon position was higher than that at the first and second positions in the protein-coding regions. And this bias can be used to discriminate cp DNA from nuclear and mitochondrial DNA [8]. SSRs are important and widely developed as molecular genetic markers for species identification [49]. Long repeat structures could promote the rearrangement of the cp genome and increase the population’s genetic diversity [50]. Hexa- nucleotides only occured in *A. villosum* among the three sequenced cp genomes, which indicates that *A. villosum* might have a more flexible cp genomes than *A. maximum* and *A. longipetiolatum*. Introns have important roles in the regulation of gene expression and can accumulate more mutations than exons; thus, they maybe contain older gene function information that has lost during evolution [51]. There were 14 genes containing one intron of *Amomum* cp genomes, meanwhile, *ycf*3 and *clp*P containing two introns that might have intron splicing events and lead to the low Ka/Ks [52].

Most of the protein-coding genes of *Amomum* species were found to be under purifying selection by Ka/Ks analysis, which was conservative in plastid genomes of most angiosperms [33]. Several CP genes under positive selection were also identified while only 1 substitution was found during the Ka/Ks calculation process for those genes, so a 3D-structure analysis was conducted to minimize the false positive results. as a result, mutations in *psb*I*, mat*K*, rps*8 *and psb*J were found to result typical structure alteration, and the hypothesis that those genes might be undergone positive selection during evolution could not be rejected. However, whether the function is actually affected still need further validation by molecular biology techniques. It seems that 3D-structures for the majority of genes with Ka/Ks > 1 are still conserved indicating most of the positively-selected genes tested with Ka/Ks > 1 by KaKs_calculator are false negative results. Additionally, the positively-selected genes with 3D-structure conserved also have been reported by other researches, such as *cem*A (Envelope membrane protein), *ndh*C (NADH-dehydrogenase) and *rps*15 (Small subunit of ribosome). *ndh*C was found undergo positive selection in other species from Zingiberaceae, such as *Amomum kravanh* [41], *Alpinia oxyphylla* [43] and *Curcuma* species [52]. Genes under positive selection might play vital roles in obtaining higher fitness to diverse environment. The *ndh* gene retains photosynthetic ability due to the non-functional role and the activity of the NDH complex seems to be of particular importance for adaptation of the photosynthetic machinery to stress conditions [53]. For these typical shade-loving plants whose natural habitats are shade forests of South China, the phototrophic component of NADH-dehydrogenase (*ndh*C) must be critical in adapting to light or other stress conditions that related to the adaptation of photosynthetic machinery at the chloroplast level [43]. In addition, our phylogenetic analysis confirmed that the paraphyletic status of species in *Amomum* and further validate the phylogenetic relationships in the Zingiberaceae reported in previous studies [1, 11].

### Species identification based on cp genomes

DNA barcoding is an efficient tool for species identification [54]; however, it doesn’t work for some closely related taxa such as *A. villosum* and *A. villosum* var. *xanthioides* as demonstrated here (Fig. S4-S19) and in previous studies [9]. Thus, the complete chloroplast genomes containing large-scale information sites are used as super barcodes or to screen more variable regions for resolution enhancement [55, 56]. In this study, we sequenced and annotated three cp genomes of *Amomum* species and conducted a comparative analysis with the published cp genome data of this genus. We tested and verified the highly variable regions of these cp genomes through experiments on 37 samples from 13 species in *Amomum*. Finally, 8 markers were mined with a better species resolution than the universal markers *mat*K, *rbc*L and *psb*A-*trn*H from cp genomes in *Amomum* species identification. We proved that specific barcodes derived from the comparison among cp genomes targeted at specific taxa could be more efficient in specific species identification instead of using a universal barcode. Compared with those highly variable sites identified by Cui et al. [8], we found different ones possibly because we used different *Amomum* species in our analyses. And adding more species might be more accurate for developing highly variable markers.

However, we found the nuclear barcodes ITS2 perform better in *Amomum* species identification than all the cpDNA barcodes including specific barcodes in this study (Fig. S4-S19 and Table 3), which is consistent with the previous study [10]. This is possibly due to the uniparental inheritance of cpDNA with low nucleotide substitution mutation rates [12]. Therefore, the use of cpDNA in species with frequent gene exchange has certain limitations. We confirmed ITS2 is an excellent DNA barcode with short fragment length, high amplification efficiency and the strongest species resolving power in our study. Our team has also constructed a reference ITS2 barcode library with 1276 sequences for Southern Chinese Medicine for its quality monitoring and control [54].

Though nuclear marker ITS2 had the strongest species identification resolution among all the assessed markers, it did not distinguish every species in *Amomum*. Instead, individuals from one species always clustered together based on the whole cp genomes (Fig. 6 and S20), indicating a better species identification trend than ITS2, yet there needs more cp genome data to support. Meanwhile, the advantage of the chloroplast genome as a super barcode for species identification was then validated. As the 3rd-generation sequencing technology represented by PacBio platform and Oxford Nanopore are becoming more and more mature, obtaining the cp genomes must be much easier and more convenient. We believe that the cp genomes will play a more important role in species identification in the future.

### Interspecific hybrids identification by ITS2 reads analysis and cp genomes

Sexual hybridization of plants belonging to different species is a naturally occurring phenomenon [57]; ITS2 could also be used to identify hybrid strains [58]. As a part fragment of rDNA with multiple copies of variation [59], holotypes of ITS2 could be fully revealed by NGS sequencing than Sanger method. In this study, ITS2 analysis was performed on the NGS data of five *A. villosum* and *A. longiligulare* samples (Fig. 8 and Table S6). The results showed that more than 94% of the reads in all samples matched the haplotype analysis by sanger sequencing. In the next-generation data, there were a few reads that could not be matched to the sanger sequencing results, which possibly be caused by the base exchange of hybrids, PCR errors, etc. For the non-hybrid samples, only the haplotype reads corresponding to the species appeared in *A. villosum* and *A. longiligulare*; for the hybrid samples, both the haplotype reads of male and female parent were present and accounted for the main proportion in each NGS data, which might also reflect the hybridization. For example, for the artificially hybridized *A. villosum* sample (CXSR), it’s possible that the hybrid progeny had undergone some hybridizations, resulting in the ratio of the ITS2 haplotypes of *A. villosum* and *A. longiligulare* in the NGS data to 1.62 while the ratio was nearly 1 in the sample SRR8185318, indicating that only one hybridization event might have occurred. Hybridization was detected simultaneously in the samples of this study and the other study (SRR8185318), suggesting that this phenomenon may be relatively common between *A. villosum* and *A. longiligulare* in nature.

In addition, it should be noted that there is the same haplotype (C-T-T-G) between *A. villosum* and *A. longiligulare* based on the 4 hybridization sites when analyzing ITS2 sequences with Sanger sequencing. But ITS2 sequences with this haplotype exhibit two extremes in the proportion of two species—the largest proportions in *A. villosum* while the least proportion in *A. longiligulare* (Table S5). Therefore, we classified the reads with this haplotype as *A. villosum* during our analysis. We are not yet able to determine whether this haplotype in *A. longiligulare* is caused by misidentification (such as a hybrid) or not. Taking a cautious approach, we recommend using the dominant haplotype sequences of *A. villosum* and *A. longiligulare* to identify hybrids, that is, when the majority of ITS2 reads from NGS data are C-T-T-G (*A. villosum*) and T-C-G-A (*A. longiligulare*), the sample could be determined as a hybrid. When primarily one of these two types, it is a purebred.

This study demonstrates that high-throughput sequencing-based ITS2 analysis could be an efficient tool for interspecific hybrid identification and with the help of the chloroplast genome, the hybrid parents can be also determined.

## Conclusions

We sequenced and annotated the complete chloroplast genomes of *A. villosum, A. maximum* and *A. longipetiolatum* by high-throughput sequencing*.* The comparative analysis was conducted for a better understanding of cp genome structures and evolutionary history of *Amomum* species. We found most of the protein-coding genes of *Amomum* species were under purifying selection. Furthermore, most of the mined genetic markers performed better than universal DNA barcodes from cp genomes in *Amomum* species identification. Phylogenetic analysis proved that *Amomum* is paraphyletic, and with the ITS2 analysis, we found the sequenced sample of *A. villosum* to be a hybrid, becoming the first report of natural hybridization of this genus. This study provides insights into species identification and evolutionary relationships of *Amomum*.

## Materials and methods

### Plant materials, DNA extraction and sequencing

Fresh and healthy leaf samples of three species *A. villosum*, *A. longipetiolatum* and *A. maximum* were collected for cp genomes sequencing. When the leaves were removed from the plants, they were stored in the liquid nitrogen in the field and were transferred to the − 80 °C refrigerator (Eppendorf, Hamburg, Germany) right back to the laboratory. 37 samples of dry leaves were collected from 13 *Amomum* species for species identification verification. All the samples were obtained from South China Botanical Garden, Chinese Academy of Sciences and identified by Ye Yushi, the engineer in SCBG. And all the voucher specimens were deposited in the Second Clinical College of Guangzhou University of Chinese Medicine (Table S2). No permission was required to collect the above samples and we confirm that all methods were performed in accordance with IUCN Policy Statement on Research Involving Species at Risk of Extinction and the Convention on the Trade in Endangered Species of Wild Fauna and Flora. We extracted total genomic DNA using a Qiagen DNeasy Plant Mini kit (Qiagen Co., Hilden, Gemany). The quality and quantity of extracted DNA was determined with 1% gel electrophoresis and Nanodrop2000C (ThermoScientific, Delaware, USA). Pure DNA from dry leaves was used in amplification of DNA markers and sanger sequencing. Pure DNA from fresh leaves was used in the cp genomes sequencing by Illumina NovaSeq (Illumina Inc., CA, USA) platform at Shanghai Majorbio Pharmaceutical Technology Co., Ltd. that generating the whole genome shotgun by Paired end library of 150 bp.

### Chloroplast genome assembly and annotation

Many strategies are available for chloroplast genome assembly [60, 61], here we referred to the method of Zhou et al. [62]. The quality of sequencing raw data was evaluated by FastQC [41] and trimmed using Trimmomatic software [63]. To extract cp-like reads from clean reads, we used BLAST searches in a reference database constructed by all cp genomes retrieved from NCBI. The extracted reads were then assembled into contigs by SOAPdenovo [22]. SSPACE [64] and Gap Filler package [65] were used to extend sequences to scaffolds and fill gaps. The complete cp genome was firstly annotated by CPGAVAS2 [23] with default parameters. For the predicted protein-coding genes, we used BLAST searches against Swiss-Prot database and checked manually in Apollo software for a more precise annotation. Then the original CPGAVAS2’s prediction was updated by the latest GFF3 file. Subsequently, tRNAs were identified by tRNAscan-SE [66]. A circular cp genome map was drawn with OGDRAW v1.3.1 (Organellar Genome DRAW) [67]. The GC content of the cp genome was calculated by GC function planted in seqinr package [68].

### Analysis of codon usage and repeat content

Relative synonymous codon usage (RSCU) in protein coding sequences of *A. villosum*, *A. longipetiolatum* and *A. maximum* was determined in CodonW. Simple sequence repeats (SSRs) of three *Amomum* species were determined by MISA [69]. For long-repeat, we downloaded other two cp genomes of *A. kravanh* (MF991963) and *A. compactum* (MG000589) from NCBI by mVISTA [70] to analyze together. Forward (F), reverse (R), complement (C) and palindromic (P) repeat types in the cp genomes were identified by the online tool REPuter [71] with default settings.

### Boundary regions and interspecific comparisons

Five cp genomes of *A. villosum*, *A. longipetiolatum*, *A. maximum*, *A. kravanh* (MF991963) and *A. compactum* (MG000589) were analyzed. The contraction and expansion of IR regions were visualized with IRScope [27] package between the four main parts of cp genomes (LSC/IRb/SSC/IRa). For interspecific comparisons, the five cp genomes were aligned using MAFFT [72]. Nucleotide diversity was calculated through sliding window analysis with DnaSP [28] based on alignment results. The step size and window length were set to 200 bp and 800 bp respectively for DnaSP.

### Species identification markers mining

The shared protein-coding genes and intergenic regions for the five *Amomum* species were extracted and calculated with Pi values. Specific primer pairs were designed at the conserved regions of gene fragments with high Pi values and amplified as the candidate markers for species identification. For comparison, the universal plant DNA barcodes including ITS, ITS2, *mat*K, *rbc*L and *psb*A*-trn*H were also amplified. A total of 37 dry leaf samples of 13 *Amomum* species were used to test and verify. The PCR reaction system of the DNA markers contained 2xTaq PCR Mix 12.5 μL, forward primer (10.0 μM) 1.0 μL, reverse primer (2.5 μM) 1.0 μL, genomic DNA 2.0 μL, and added up to 25 μL with ddH_2_O. The primers and conditions for PCR were listed in Table S7. All the PCR products were sent to Sangon Biotech Guangzhou branch office for sequencing. The bi-directionally sequenced peaks of DNA markers were assembled using the CondonCode Aligner v8.0.1 software. Maximum Likelihood (ML) trees were constructed with 1000 bootstrap replicates for each marker in MEGA version 6.0 [73] to evaluate its discrimination power [substitution model], using 50% as a cut-off value for the condensed tree. Sequence length, variable sites and K2P distances were statistics by MEGA.

### Selective pressure estimation and phylogenetic analysis

We extracted shared non-redundant gene CDS among 20 cp genomes of 12 species from *Amomum* and *Alpinia*, each gene’s CDS-pair of one-by-one species’ combination were extracted and were aligned by MAFFT. The rates of synonymous substitutions (Ks) and non-synonymous substitutions (Ka) and Ka/Ks were then calculated by ParaAT2.0 [74] which is planted in KaKs_Calculator2.0 with ‘MA’ model [30], the command we applied in this study is as follows: “ParaAT.pl -c 11 -h homologs.txt -n CDS -a PEP -p proc -o OUT -k -f axt -m mafft -v”. The 37 cp genome sequences from 20 species were aligned by MAFFT. And the alignment results were used to construct Bayesian, maximum likelihood (ML) and neighbor joining (NJ) trees with MrBayes3.2 [75], RAxML-ng [34] with and MEGA [76] respectively. The parameters for MrBayes. Default parameters of MrBayes were used in this study; The parameters for RAxML-ng were set as follows: GTR with 4 free rates heterogeneity but with ML estimate of the base frequencies model (GTR + R4 + FO) was choosed, default tree searching strategy was choosed using 10 random and 10 parsimony-based starting trees (tree = pars, rand), Transfer Bootstrap Expectation (TBE) and standard booststrap support metric (Felsenstein’s bootsrap, FBP) were applied (bs-metric = fbp, tbe) [77]. 100 starting trees (50 random and 50 parsimony-based) were used to pick the best-scoring topology, bootstrap replicates were set to 1000. Parameters for MEGA were set as follows: bootstrap value to 10,000, Kimura 2-parameter model were choosed for nucleotide substitution, Neighbor-joining method were applied to construct NJ trees.

### ITS2 analysis

We downloaded all the ITS2 sequences of *A. villosum* and *A. longiligulare* from NCBI. The sequences were aligned with MEGA version 6.0 [73]. For the ITS2 analysis of NGS data, Bowtie2 [35] was used to align the sequencing reads to ITS2 of *A. villosum*. Then the files were sorted and formatted by Samtools [78], and observed by IGV. The variable sites were counted by the written JavaScript. The reads that not fully covered the four sites were neglected. For validation, we also analyzed the downloaded NGS data of *A. villosum* (SRR8185318) and *A. longiligulare* (SRR10769494) from NCBI, and the received NGS data from Xiasheng Zheng (*A. villosum*, CGSR), Danyan Zhang and Shijie Li (artificially hybridized sample of *A. villosum*, CXSR).

## Supplementary Information

**Additional file 1: Table S2.**. Genbank accession numbers of DNA barcodes. **Table S4**. Results for one-vs-one calculation of Ka/Ks generated by KaKs_Calculator.

**Additional file 2: Table S1.**. Number of different SSR types detected in three *Amomum* species. **Table S3**. Species information of Ka/Ks. **Table S5.** ITS2 haplotypes of *A. villosum* and *A. longiligulare* from Sanger sequencing. **Table S6**. ITS2 reads of *A. villosum* and *A. longiligulare* from NGS data. **Table S7.** The primers and conditions for PCR.

**Additional file 3: Fig. S1.** Analysis of simple sequence repeats (SSRs) in the cp genomes of three *Amomum* species. **Fig. S2**. Frequency of identified SSR motifs in different repeat class types. **Fig. S3.** Repeat sequences of three *Amomum* species. F, P, R, and C indicate the repeat types F (forward), P (palindrome), R (reverse), and C (complement), respectively. **Fig. S4.** ML tree based on ccsA sequences of *Amomum* species. This Bootstrap consensus tree was constructed by K2P model with 1000 bootstrap replicates. Numbers under the nodes indicate bootstrap probabilities. The cut off value for the condensed tree was 50%. **Fig. S5.** ML tree based on *trn*C-*GCA*_*pet*N sequences of *Amomum* species. This Bootstrap consensus tree was constructed by K2P model with 1000 bootstrap replicates. Numbers under the nodes indicate bootstrap probabilities. The cut off value for the condensed tree was 50%. **Fig. S6.** ML tree based on *ndh*B_*rps*7 sequences of *Amomum* species. This Bootstrap consensus tree was constructed by K2P model with 1000 bootstrap replicates. Numbers under the nodes indicate bootstrap probabilities. The cut off value for the condensed tree was 50%. **Fig. S7.** ML tree based on *psa*I_*ycf*4 sequences of *Amomum* species. This Bootstrap consensus tree was constructed by K2P model with 1000 bootstrap replicates. Numbers under the nodes indicate bootstrap probabilities. The cut off value for the condensed tree was 50%. **Fig. S8.** ML tree based on *rpl*20 sequences of *Amomum* species. This Bootstrap consensus tree was constructed by K2P model with 1000 bootstrap replicates. Numbers under the nodes indicate bootstrap probabilities. The cut off value for the condensed tree was 50%. **Fig. S9.** ML tree based on *rpl*33 sequences of *Amomum* species. This Bootstrap consensus tree was constructed by K2P model with 1000 bootstrap replicates. Numbers under the nodes indicate bootstrap probabilities. The cut off value for the condensed tree was 50%. **Fig. S10.** ML tree based on *rps*3 sequences of *Amomum* species. This Bootstrap consensus tree was constructed by K2P model with 1000 bootstrap replicates. Numbers under the nodes indicate bootstrap probabilities. The cut off value for the condensed tree was 50%. **Fig. S11.** ML tree based on* rpo*A sequences of *Amomum* species. This Bootstrap consensus tree was constructed by K2P model with 1000 bootstrap replicates. Numbers under the nodes indicate bootstrap probabilities. The cut off value for the condensed tree was 50%. **Fig. S12.** ML tree based on *rps*4 sequences of *Amomum* species. This Bootstrap consensus tree was constructed by K2P model with 1000 bootstrap replicates. Numbers under the nodes indicate bootstrap probabilities. The cut off value for the condensed tree was 50%. **Fig. S13.** ML tree based on *ndh*D_1 sequences of *Amomum* species. This Bootstrap consensus tree was constructed by K2P model with 1000 bootstrap replicates. Numbers under the nodes indicate bootstrap probabilities. The cut off value for the condensed tree was 50%. **Fig. S14.** ML tree based on *ndh*D_2 sequences of *Amomum* species. This Bootstrap consensus tree was constructed by K2P model with 1000 bootstrap replicates. Numbers under the nodes indicate bootstrap probabilities. The cut off value for the condensed tree was 50%. **Fig. S15.** ML tree based on ITS sequences of *Amomum* species. This Bootstrap consensus tree was constructed by K2P model with 1000 bootstrap replicates. Numbers under the nodes indicate bootstrap probabilities. The cut off value for the condensed tree was 50%. **Fig. S16.** ML tree based on ITS2 sequences of *Amomum* species. This Bootstrap consensus tree was constructed by K2P model with 1000 bootstrap replicates. Numbers under the nodes indicate bootstrap probabilities. The cut off value for the condensed tree was 50%. **Fig. S17.** ML tree based on *psb*A-*trn*H sequences of *Amomum* species. This Bootstrap consensus tree was constructed by K2P model with 1000 bootstrap replicates. Numbers under the nodes indicate bootstrap probabilities. The cut off value for the condensed tree was 50%. **Fig. S18.** ML tree based on *mat*K sequences of *Amomum* species. This Bootstrap consensus tree was constructed by K2P model with 1000 bootstrap replicates. Numbers under the nodes indicate bootstrap probabilities. The cut off value for the condensed tree was 50%. **Fig. S19.** ML tree based on *rbc*L sequences of *Amomum* species. This Bootstrap consensus tree was constructed by K2P model with 1000 bootstrap replicates. Numbers under the nodes indicate bootstrap probabilities. The cut off value for the condensed tree was 50%. **Fig. S20.** 3D-structure for the represented positively-selected genes with 1 substitution. **Fig. S21**. Phylogenetic tree constructed by NJ based on complete chloroplast genome. Numbers under the nodes indicate bootstrap probabilities. Our sequenced samples are marked in red. Species from different genuses are marked in different color on the nodes.

## Data Availability

All original data is available from the NCBI database. The accession numbers of chloroplast genome sequences of *A. villosum, A. longipetiolatum* and *A. maximum* are MW970344, MW995975 and MW995976. The accession numbers of sequences amplified from cp genomes with universal and specific primers are listed in Table S2.

## Abbreviations

Cp: Chloroplast
LSC: Large single-copy region
IR: Inverted repeat region
SSC: Small single-copy region
CDS: Coding DNA sequence
tRNAs: Transport RNAs
rRNAs: Ribosomal RNAs
SSRs: Simple sequence repeats
Pi: Nucleotide diversity
Ka/Ks: The rate of non-synonymous substitutions to the rate of synonymous substitutions
RSCU: Relative synonymous codon usage
