# Supplementary material for: Comparative chloroplast genome analyses of Amomum: insights into evolutionary history and species identification
Source: BMC Plant Biol. 2022 Nov 9;22:520. doi: 10.1186/s12870-022-03898-x (PMC9644571; doi:10.1186/s12870-022-03898-x)
Supplement: Supplementary file 2 — Additional file 2: Table S1.. Number of different SSR types detected in three Amomum species. Table S3. Species information of Ka/Ks. Table S5. ITS2 haplotypes of A. villosum and A. longiligulare from Sanger sequencing. Table S6. ITS2 reads of A. villosum and A. longiligulare from NGS data. Table S7. The primers and conditions for PCR. [file 12870_2022_3898_MOESM2_ESM.docx]

**Table S1 Number of different SSR types detected in three *Amomum* species**

|  | *A.villosum* | *A.longipetiolatum* | *A.maximum* |
| --- | --- | --- | --- |
| Mononucleotide | 90 | 50 | 63 |
| Dinucleotide | 33 | 35 | 35 |
| Trinucleotide | 6 | 6 | 5 |
| Tetranucleotide | 16 | 18 | 24 |
| Pentanucleotide | 5 | 4 | 3 |
| Hexanucleotide | 2 | 0 | 0 |
| Total | 152 | 113 | 130 |

**Table S3 Species information of Ka/Ks**

| ID | Species | ACC_Num | Abbreviation | Remarks |
| --- | --- | --- | --- | --- |
| 1 | *Amomum kravanh* | MF991963 | Akr |  |
| 2 | *Amomum compactum* | MG000589 | Aco |  |
| 3 | *Amomum villosum* | MK262730 | Avi_1 |  |
| 4 | *Amomum villosum* | MH161418 | Avi_2 |  |
| 5 | *Amomum villosum* | MH161416 | Avi_3 |  |
| 6 | *Amomum villosum* | MN067431 | Avi_4 |  |
| 7 | *Amomum villosum* | MK389642 | Avi_6 |  |
| 8 | *Amomum villosum* Lour. var*. xanthioides* | MN067433 | AviL_1 |  |
| 9 | *Amomum villosum* Lour. var*.* *xanthioides* | MH161417 | AviL_2 | HNS |
| 10 | *Amomum longiligular* | MN067434 | Alog_1 |  |
| 11 | *Amomum longiligular* | MN067435 | Alog_2 |  |
| 12 | *Amomum longiligular* | MK889505 | Alog_3 |  |
| 13 | *Alpinia zerumber* | JX088668 | Aze | outgroup |
| 14 | *Alpinia oxyphylla* | NC_035895.1 | Aox_1 | outgroup |
| 15 | *Alpinia oxyphylla* | MK262729 | Aox_2 | outgroup |
| 16 | *Alpinia katsumadai* | MK262728 | Aka | outgroup |
| 17 | *Alpinia pumila* | MK262731 | Apu | outgroup |
| 18 | *Amomum longipetiolatum* | MW995975 | Alop | CBD |
| 19 | *Amomum maximum* | MW995976 | Ama | JSR |
| 20 | *Amomum villosum* | MW970344 | Avi | SR |

**Table S5 ITS2 haplotypes of *A. villosum* and *A. longiligulare* from Sanger sequencing**

| Species | Haplotypes | No. of sequences | Percentage |
| --- | --- | --- | --- |
| *A. villosum* | C-T-T-G | 22 | 70.97 |
|  | T-C-G-G | 5 | 16.13 |
|  | C-C-T-G | 2 | 6.45 |
|  | C-C-G-G | 2 | 6.45 |
| *A. longiligulare* | T-C-G-A | 19 | 82.61 |
|  | T-T-G-G | 3 | 13.04 |
|  | C-T-T-G | 1 | 4.35 |

**Table S6 ITS2 reads of *A. villosum* and *A. longiligulare* from NGS data**

| Smples | Haplotypes | Matches to species | No. of reads | Percentage |
| --- | --- | --- | --- | --- |
| Our sample  (MW995976) | C-T-T-G | *A. villosum* | 669 | 47.41 |
|  | T-C-G-A | *A. longiligulare* | 585 | 41.46 |
|  | T-C-G-G | *A. villosum* | 76 | 5.39 |
|  | C-T-A-G | none | 56 | 3.97 |
|  | C-T-G-G | none | 9 | 0.64 |
|  | T-T-G-G | *A. longiligulare* | 5 | 0.35 |
|  | T-A-G-A | none | 2 | 0.14 |
|  | T-T-T-G | none | 2 | 0.14 |
|  | T-T-G-A | none | 2 | 0.14 |
|  | T-G-G-A | none | 1 | 0.07 |
|  | C-T-T-A | none | 1 | 0.07 |
|  | A-G-T-A | none | 1 | 0.07 |
|  | A-T-T-G | none | 1 | 0.07 |
|  | T-G-G-G | none | 1 | 0.07 |
| CGSR | C-T-T-G | *A. villosum* | 684 | 95.66 |
|  | C-T-G-G | none | 13 | 1.82 |
|  | T-T-T-G | none | 12 | 1.68 |
|  | C-T-T-A | none | 3 | 0.42 |
|  | G-T-T-G | none | 2 | 0.28 |
|  | C-T-C-G | none | 1 | 0.14 |
| SRR8185318 | C-T-T-G | *A. villosum* | 836 | 45.14 |
|  | T-C-G-A | *A. longiligulare* | 846 | 45.68 |
|  | T-C-G-G | *A. villosum* | 84 | 4.54 |
|  | C-T-A-G | none | 61 | 3.29 |
|  | C-T-G-G | none | 8 | 0.43 |
|  | T-T-G-A | none | 3 | 0.16 |
|  | T-T-G-G | *A. longiligulare* | 3 | 0.16 |
|  | T-T-T-G | none | 3 | 0.16 |
|  | C-C-T-G | *A. villosum* | 1 | 0.05 |
|  | A-T-A-G | none | 1 | 0.05 |
|  | T-C-G-C | none | 1 | 0.05 |
|  | C-T-T-A | none | 1 | 0.05 |
|  | T-G-G-A | none | 1 | 0.05 |
|  | G-C-G-A | none | 1 | 0.05 |
|  | N-C-G-A | none | 1 | 0.05 |
|  | T-A-G-A | none | 1 | 0.05 |
| CXSR | C-T-T-G | *A. villosum* | 427 | 53.78 |
|  | T-C-G-A | *A. longiligulare* | 292 | 36.78 |
|  | T-C-G-G | *A. villosum* | 50 | 6.30 |
|  | C-T-G-G | none | 9 | 1.13 |
|  | C-G-T-G | none | 3 | 0.38 |
|  | T-T-T-G | none | 2 | 0.25 |
|  | T-T-G-G | *A. longiligulare* | 2 | 0.25 |
|  | C-T-C-G | none | 2 | 0.25 |
|  | C-T-T-T | none | 1 | 0.13 |
|  | T-G-G-G | none | 1 | 0.13 |
|  | T-A-G-G | none | 1 | 0.13 |
|  | C-T-T-C | none | 1 | 0.13 |
|  | G-C-G-A | none | 1 | 0.13 |
|  | T-C-C-A | none | 1 | 0.13 |
|  | T-A-G-A | none | 1 | 0.13 |
| SRR10769494 | T-C-G-A | *A. longiligulare* | 76 | 98.70 |
|  | T-C-G-C | none | 1 | 1.30 |

**Table S7 The primers and conditions for PCR**

| **No.** | **Primer** | **Base (5’-3’)** | **PCR amplification conditions** |
| --- | --- | --- | --- |
| 1 | ITS2 2F  3R | ATGCGATACTTGGTGTGAAT  GACGCTTCTCCAGACTACAAT | 94℃，5min；94℃，30s，56℃，30s，72℃，45s，40 circles；72℃，10min |
| 2 | ITS 5F  4R | CCTTATCATTTAGAGGAAGGAG  TCCTCCGCTTATTGATATGC | 94℃，5min；94℃，1min，50℃，1min，72℃，1min30s，30 circles；72℃，7min |
| 3 | *psb*A*-trn*H fwd PA  rev TH | GTTATGCATGAACGTAATGCTC  CGCGCATGGTGGATTCACAATCC | 94℃，5min；94℃，1min，55℃，1min，72℃，1.5min，30 circles；72℃，7min |
| 4 | *mat*K 3F_KIM  1R_KIM | CGTACAGTACTTTTGTGTTTACGAG  ACCCAGTCCATCTGGAAATCTTGGTTC | 94℃，1min；94℃，30s，52℃，20s，72℃，50s，35 circles；72℃，5min |
| 5 | *rbc*L 1f  724r | ATGTCACCACAAACAGAAAC  TCGCATGTACCTGCAGTAGC | 95℃，2min；94℃，1min，55℃，30s，72℃，1min，34 circles；72℃，7min |
| 6 | *ndh*B*_rps*7 F  R | GAATTAGTAACTGCATCCAATCT  TCCATGAGCAGGATCTATATAGACAC | 94℃，5min；94℃，30s，57℃，1min，72℃，1min15s，40 circles；72℃，10min |
| 7 | *psa*I*_ycf*4 F  R | ATGGTAATGAATCTTATCAAGTG  ACTATACTGCATTCAGTTGCAT | 94℃，5min；94℃，30s，55℃，45s，72℃，1min15s，40 circles；72℃，10min |
| 8 | *trn*C*-GCA_pet*N F  R | CCTAGGATTGAAGAAGAGATTG  AATAATCATAGTGGAATTAATGGC | 94℃，5min；94℃，30s，55℃，45s，72℃，1min15s，40 circles；72℃，10min |
| 9 | *rpl*20 F  R | TCGTTTGTGGATCACTCGGA  CCGGGGAATTCTGTTTCGTT | 94℃，5min；94℃，30s，56℃，1min，72℃，1min15s，40 circles；72℃，10min |
| 10 | *rpl33* F  R | GTAAGGATGTAAGAGTTATAGTT  AATCGTATGCTTGTGACAATAG | 94℃，5min；94℃，30s，55℃，45s，72℃，1min15s，40 circles；72℃，10min |
| 11 | *ccs*A F  R | CAATTGTTGAACATATATTGACTC  GTAGATGTTAATGTGAATGAACC | 94℃，5min；94℃，30s，57℃，1min，72℃，1min15s，40 circles；72℃，10min |
| 12 | *rps*3 F  R | AGACTTGGTACAACTCAACATC  ATAGTTCGAATTATATAGGAACAA | 94℃，5min；94℃，30s，57℃，1min，72℃，1min15s，40 circles；72℃，10min |
| 13 | *rpo*A F  R | GTGTTGAATCAAGAATAGATAGTA  ATATTCAATTCTCATAAGATCTTC | 94℃，5min；94℃，30s，57℃，1min，72℃，1min15s，40 circles；72℃，10min |
| 14 | *rps*4 F  R | TGTCTCGTTACCGAGGACC  ATAATATTCTACGACTAACAACTC | 94℃，5min；94℃，30s，57℃，1min，72℃，1min15s，40 circles；72℃，10min |
| 15 | *ndh*D_1 F  R | CCTTGGCTAACAATACTTGTTG  CAGCTAGAAGCATACATGTACT | 94℃，5min；94℃，30s，57℃，1min，72℃，1min15s，40 circles；72℃，10min |
| 16 | *ndh*D_2 F  R | AGTACATGTATGCTTCTAGCTG  TAGTTCTCGTGGTCCAGAATC | 94℃，5min；94℃，30s，57℃，1min，72℃，1min15s，40 circles；72℃，10min |
