# Supplementary material for: Comparative chloroplast genome analyses of Amomum: insights into evolutionary history and species identification
Source: BMC Plant Biol. 2022 Nov 9;22:520. doi: 10.1186/s12870-022-03898-x (PMC9644571; doi:10.1186/s12870-022-03898-x)
Supplement: Supplementary file 3 — Additional file 3: Fig. S1. Analysis of simple sequence repeats (SSRs) in the cp genomes of three Amomum species. Fig. S2. Frequency of identified SSR motifs in different repeat class types. Fig. S3. Repeat sequences of three Amomum species. F, P, R, and C indicate the repeat types F (forward), P (palindrome), R (reverse), and C (complement), respectively. Fig. S4. ML tree based on ccsA sequences of Amomum species. This Bootstrap consensus tree was constructed by K2P model with 1000 bootstrap replicates. Numbers under the nodes indicate bootstrap probabilities. The cut off value for the condensed tree was 50%. Fig. S5. ML tree based on trnC-GCA_petN sequences of Amomum species. This Bootstrap consensus tree was constructed by K2P model with 1000 bootstrap replicates. Numbers under the nodes indicate bootstrap probabilities. The cut off value for the condensed tree was 50%. Fig. S6. ML tree based on ndhB_rps7 sequences of Amomum species. This Bootstrap consensus tree was constructed by K2P model with 1000 bootstrap replicates. Numbers under the nodes indicate bootstrap probabilities. The cut off value for the condensed tree was 50%. Fig. S7. ML tree based on psaI_ycf4 sequences of Amomum species. This Bootstrap consensus tree was constructed by K2P model with 1000 bootstrap replicates. Numbers under the nodes indicate bootstrap probabilities. The cut off value for the condensed tree was 50%. Fig. S8. ML tree based on rpl20 sequences of Amomum species. This Bootstrap consensus tree was constructed by K2P model with 1000 bootstrap replicates. Numbers under the nodes indicate bootstrap probabilities. The cut off value for the condensed tree was 50%. Fig. S9. ML tree based on rpl33 sequences of Amomum species. This Bootstrap consensus tree was constructed by K2P model with 1000 bootstrap replicates. Numbers under the nodes indicate bootstrap probabilities. The cut off value for the condensed tree was 50%. Fig. S10. ML tree based on rps3 sequences of Amomum species. This [file 12870_2022_3898_MOESM3_ESM.docx]

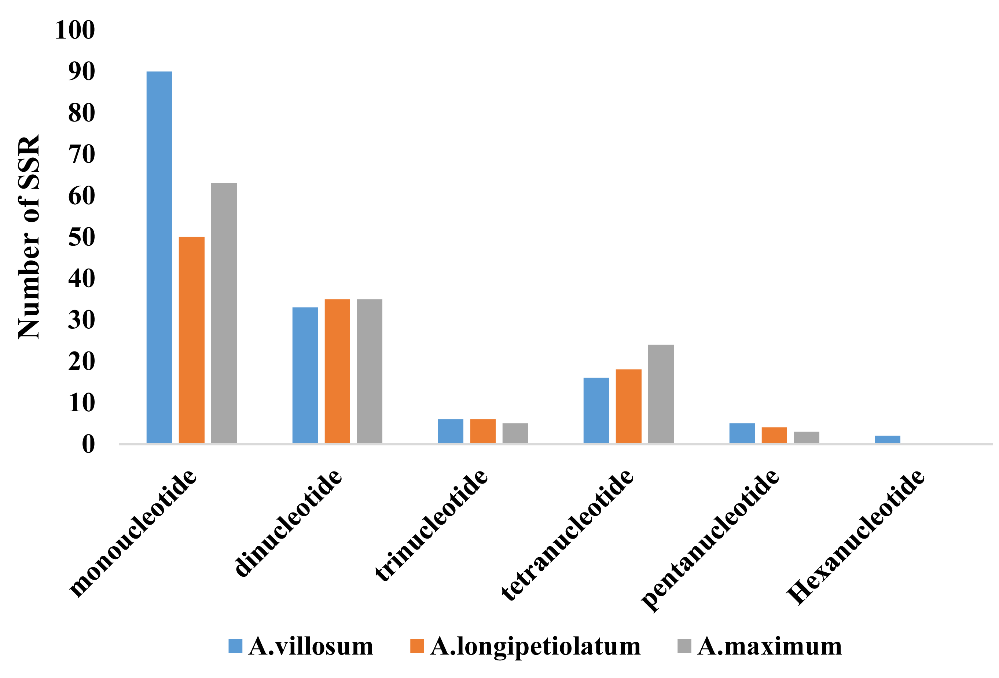


**Figure S1 Analysis of simple sequence repeats (SSRs) in the cp genomes of three *Amomum* species.**

**
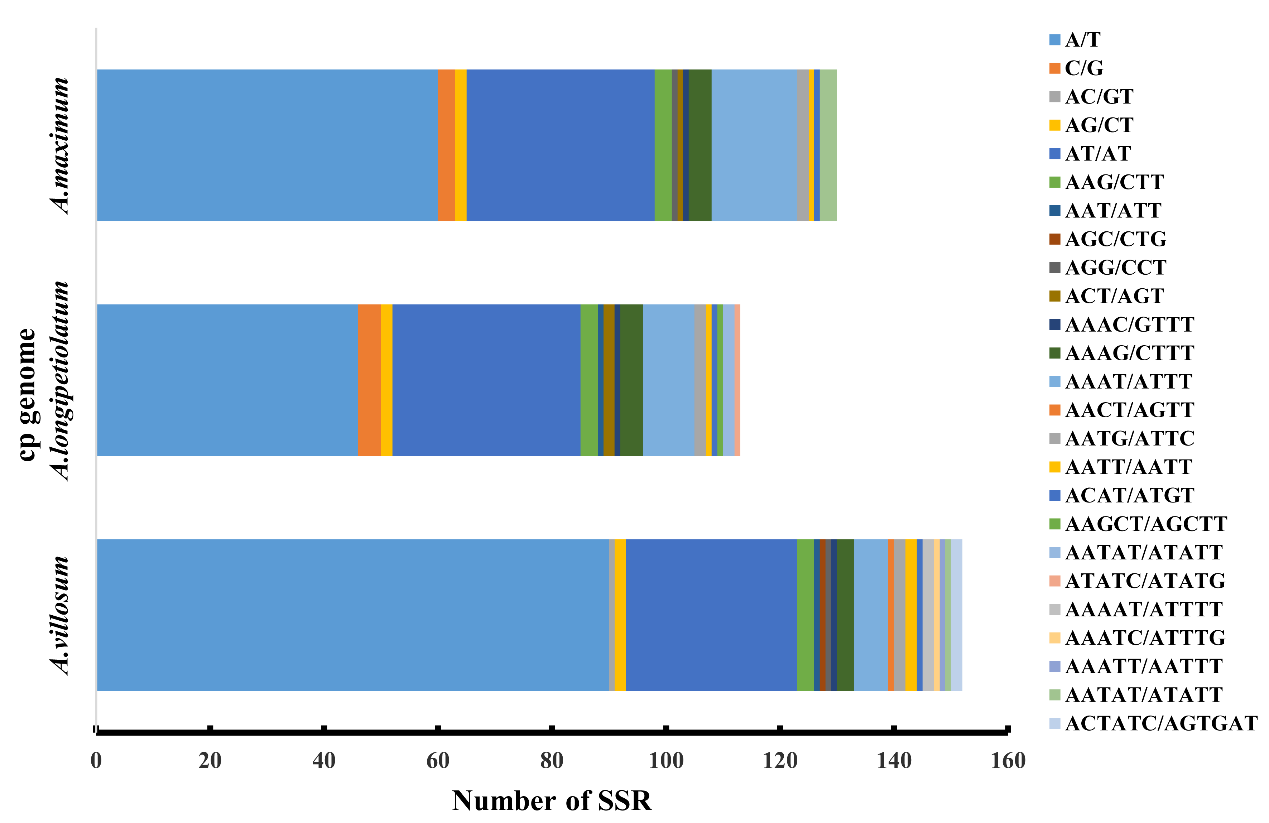
**

**Figure S2 Frequency of identified SSR motifs in different repeat class types.**

***
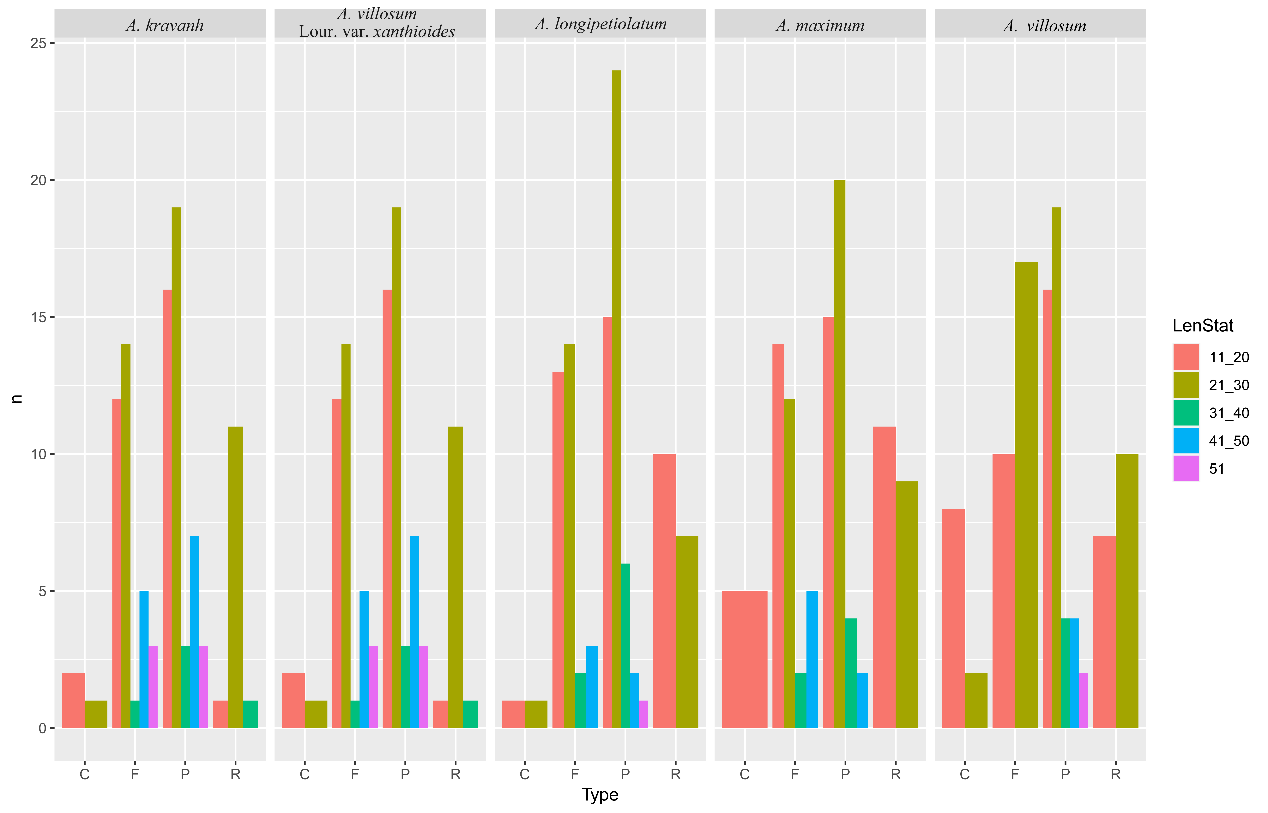
***

**Figure S3 Repeat sequences of three *Amomum* species. F, P, R, and C indicate the repeat types F (forward), P (palindrome), R (reverse), and C (complement), respectively.**

**Figure S4 ML tree based on *ccs*A sequences of *Amomum* species.** This Bootstrap consensus tree was constructed by K2P model with 1000 bootstrap replicates. Numbers under the nodes indicate bootstrap probabilities. The cut off value for the condensed tree was 50%.

**Figure S5 ML tree based on *trn*C*-GCA_pet*N sequences of *Amomum* species.** This Bootstrap consensus tree was constructed by K2P model with 1000 bootstrap replicates. Numbers under the nodes indicate bootstrap probabilities. The cut off value for the condensed tree was 50%.

**Figure S6 ML tree based on *ndh*B*_rps*7 sequences of** ***Amomum* species.** This Bootstrap consensus tree was constructed by K2P model with 1000 bootstrap replicates. Numbers under the nodes indicate bootstrap probabilities. The cut off value for the condensed tree was 50%.

**Figure S7 ML tree based on *psa*I*_ycf*4 sequences of** ***Amomum* species.** This Bootstrap consensus tree was constructed by K2P model with 1000 bootstrap replicates. Numbers under the nodes indicate bootstrap probabilities. The cut off value for the condensed tree was 50%.

**Figure S8 ML tree based on *rpl*20 sequences of** ***Amomum* species.** This Bootstrap consensus tree was constructed by K2P model with 1000 bootstrap replicates. Numbers under the nodes indicate bootstrap probabilities. The cut off value for the condensed tree was 50%.

**Figure S9 ML tree based on *rpl*33 sequences of** ***Amomum* species.** This Bootstrap consensus tree was constructed by K2P model with 1000 bootstrap replicates. Numbers under the nodes indicate bootstrap probabilities. The cut off value for the condensed tree was 50%.

**Figure S10 ML tree based on *rps*3 sequences of** ***Amomum* species.** This Bootstrap consensus tree was constructed by K2P model with 1000 bootstrap replicates. Numbers under the nodes indicate bootstrap probabilities. The cut off value for the condensed tree was 50%.

**Figure S11 ML tree based on *rpo*A sequences of** ***Amomum* species.** This Bootstrap consensus tree was constructed by K2P model with 1000 bootstrap replicates. Numbers under the nodes indicate bootstrap probabilities. The cut off value for the condensed tree was 50%.

**Figure S12 ML tree based on *rps*4** s**equences of** ***Amomum* species.** This Bootstrap consensus tree was constructed by K2P model with 1000 bootstrap replicates. Numbers under the nodes indicate bootstrap probabilities. The cut off value for the condensed tree was 50%.

**Figure S13 ML tree based on *ndh*D_1 sequences of** ***Amomum* species.** This Bootstrap consensus tree was constructed by K2P model with 1000 bootstrap replicates. Numbers under the nodes indicate bootstrap probabilities. The cut off value for the condensed tree was 50%.

**Figure S14 ML tree based on *ndh*D_2 sequences of** ***Amomum* species.** This Bootstrap consensus tree was constructed by K2P model with 1000 bootstrap replicates. Numbers under the nodes indicate bootstrap probabilities. The cut off value for the condensed tree was 50%.

**Figure S15 ML tree based on** **ITS** s**equences of** ***Amomum* species.** This Bootstrap consensus tree was constructed by K2P model with 1000 bootstrap replicates. Numbers under the nodes indicate bootstrap probabilities. The cut off value for the condensed tree was 50%.

**Figure S16 ML tree based on ITS2 sequences of** ***Amomum* species.** This Bootstrap consensus tree was constructed by K2P model with 1000 bootstrap replicates. Numbers under the nodes indicate bootstrap probabilities. The cut off value for the condensed tree was 50%.

**Figure S17 ML tree based on *psb*A*-trn*H sequences of** ***Amomum* species.** This Bootstrap consensus tree was constructed by K2P model with 1000 bootstrap replicates. Numbers under the nodes indicate bootstrap probabilities. The cut off value for the condensed tree was 50%.

**Figure S18 ML tree based o**n ***mat*K sequences of** ***Amomum* species.** This Bootstrap consensus tree was constructed by K2P model with 1000 bootstrap replicates. Numbers under the nodes indicate bootstrap probabilities. The cut off value for the condensed tree was 50%.

**Figure S19 ML tree based on *rbc*L sequences of** ***Amomum* species.** This Bootstrap consensus tree was constructed by K2P model with 1000 bootstrap replicates. Numbers under the nodes indicate bootstrap probabilities. The cut off value for the condensed tree was 50%.

**
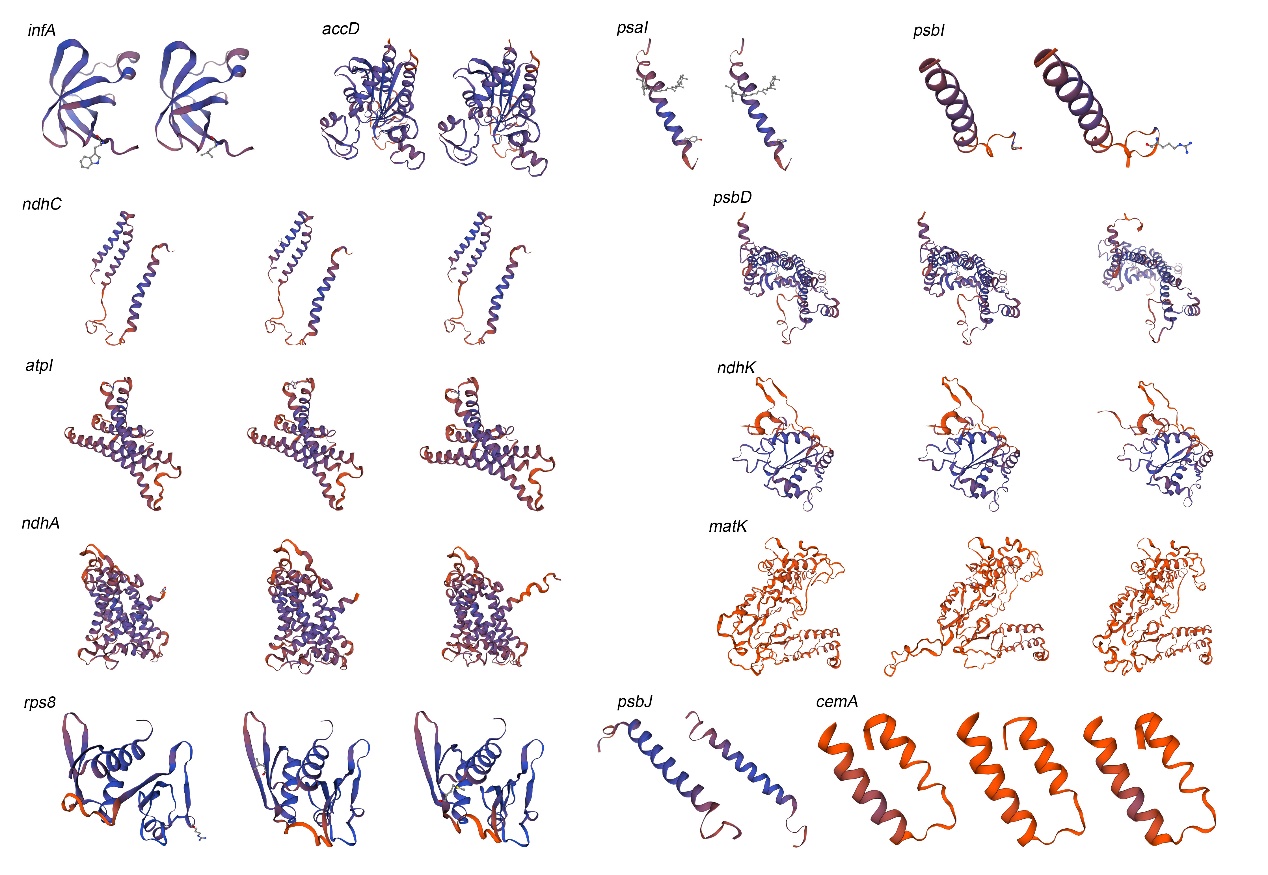
**

**Figure S20 3D-structure for the represented positively-selected genes with 1 substitution.**

**
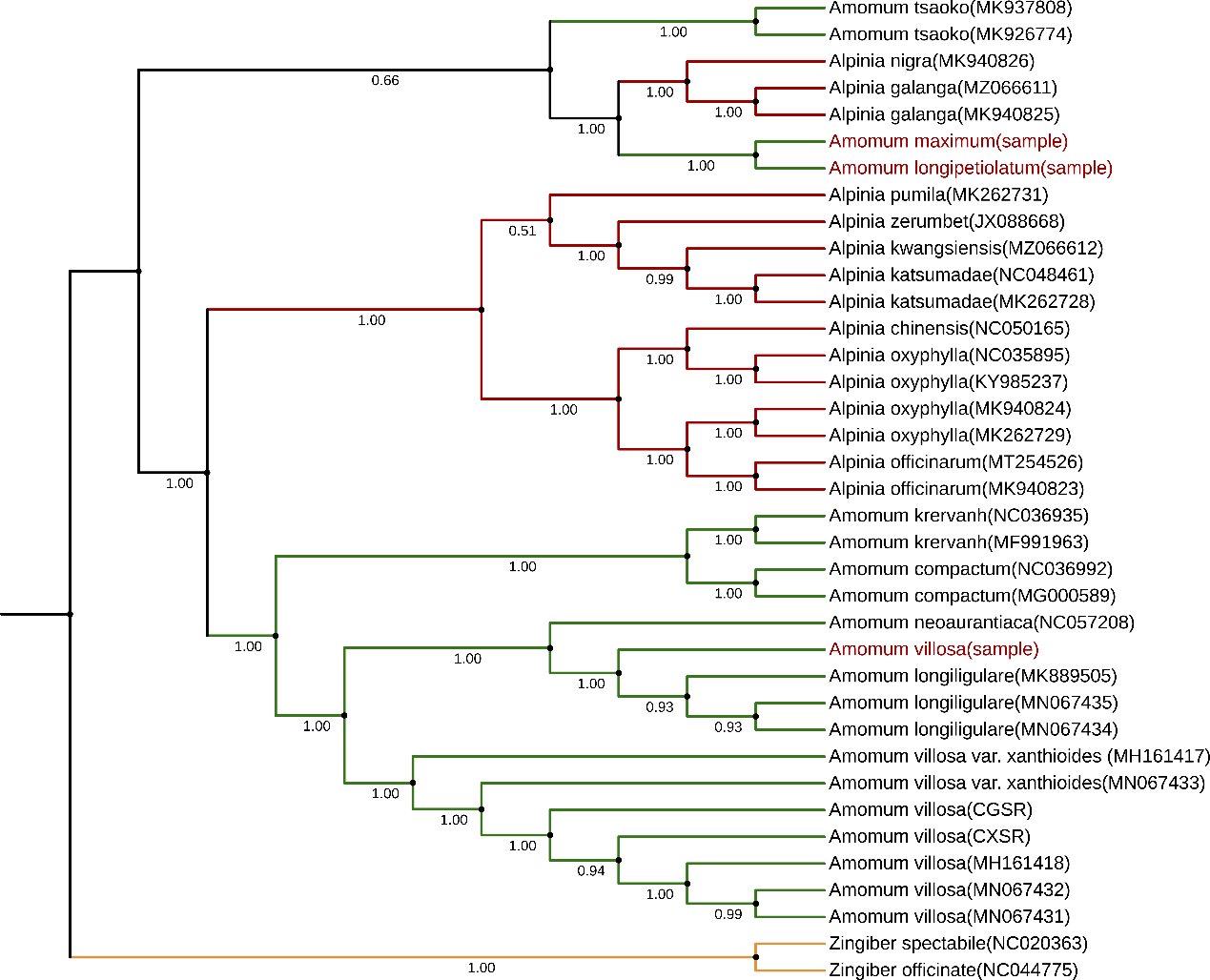
**

**Figure S21 Phylogenetic tree constructed by NJ based on complete chloroplast genome.** Numbers under the nodes indicate bootstrap probabilities. Our sequenced samples are marked in red. Species from different genuses are marked in different color on the nodes.
